# The *GBA1* D409V mutation exacerbates synuclein pathology to differing extents in two alpha-synuclein models

**DOI:** 10.1242/dmm.049192

**Published:** 2022-05-25

**Authors:** Nicole K. Polinski, Terina N. Martinez, Sylvie Ramboz, Michael Sasner, Mark Herberth, Robert Switzer, Syed O. Ahmad, Lee J. Pelligrino, Sean W. Clark, Jacob N. Marcus, Sean M. Smith, Kuldip D. Dave, Mark A. Frasier

**Affiliations:** 1 The Michael J. Fox Foundation for Parkinson's Research, Grand Central Station PO Box 4777, New York, NY 10163, USA; 2 PsychoGenics, Inc, 215 College Road, Paramus, NJ 07652, USA; 3 The Jackson Laboratory, 600 Main Street, Bar Harbor, ME 04609, USA; 4 Charles River Laboratories, 1407 George Road, Ashland, OH 44805, USA; 5 NeuroScience Associates, 10915 Lake Ridge Drive, Knoxville, TN 37934, USA; 6 Saint Louis University, 3437 Caroline Street, St. Louis, MO 63104, USA; 7 Amicus Therapeutics, 1 Cedarbrook Dr, Cranbury, NJ 08512, USA; 8 Merck & Co., Inc., 2000 Galloping Hill Road, Kenilworth, NJ 07033, USA

**Keywords:** GCase, Alpha-synuclein, Parkinson's disease

## Abstract

Heterozygous mutations in the *GBA1* gene – encoding lysosomal glucocerebrosidase (GCase) – are the most common genetic risk factors for Parkinson's disease (PD). Experimental evidence suggests a correlation between decreased GCase activity and accumulation of alpha-synuclein (aSyn). To enable a better understanding of the relationship between aSyn and GCase activity, we developed and characterized two mouse models that investigate aSyn pathology in the context of reduced GCase activity. The first model used constitutive overexpression of wild-type human aSyn in the context of the homozygous GCase activity-reducing D409V mutant form of *GBA1*. Although increased aSyn pathology and grip strength reductions were observed in this model, the nigrostriatal system remained largely intact. The second model involved injection of aSyn preformed fibrils (PFFs) into the striatum of the homozygous *GBA1* D409V knock-in mouse model. The *GBA1* D409V mutation did not exacerbate the pathology induced by aSyn PFF injection. This study sheds light on the relationship between aSyn and GCase in mouse models, highlighting the impact of model design on the ability to model a relationship between these proteins in PD-related pathology.

## Abstract (precis)

**Summary:** Phenotyping data for two novel mouse models combining glucocerebrosidase (GCase) deficiencies and alpha-synuclein pathology reveal the extent to which alpha-synuclein pathology is exacerbated by GCase deficiency.

## INTRODUCTION

Over 300 mutations in the *GBA1* gene (also known as *GBA*) encoding the lysosomal enzyme glucocerebrosidase (GCase) have been identified to date, together constituting the greatest genetic risk factor for Parkinson's disease (PD) (Rosenbloom et al., 2011; Sidransky et al., 2009). PD patients harboring mutations in *GBA1* exhibit varied phenotypes based on the severity of the mutation, but generally present with a slightly earlier age of onset and greater prevalence of cognitive changes compared to patients without *GBA1* mutations (Alcalay et al., 2010; Nichols et al., 2009; Neumann et al., 2009; Sidransky et al., 2009). The pathology between *GBA1* mutation carriers with PD and PD patients who do not harbor mutations in the *GBA1* gene is strikingly similar – including nigrostriatal degeneration and Lewy body pathology with aggregation and phosphorylation of the alpha-synuclein (aSyn) protein (encoded by *SNCA*) (Blandini et al., 2018; Goker-Alpan et al., 2010; Neumann et al., 2009). In addition, decreased GCase activity has been reported in PD patients with and without *GBA1* mutations (Mazzulli et al., 2011; Gegg et al., 2012; Murphy and Halliday, 2014; Ortega et al., 2016; Atashrazm et al., 2018). Owing to the high prevalence of *GBA1* mutations among PD patients and the similarity of pathology between *GBA1* mutation-associated PD and sporadic PD, investigating the biological underpinnings of the pathological changes associated with *GBA1* mutations and developing GCase-targeted therapeutics are areas of development for PD research.

To enable this research, multiple rodent models harboring decreased GCase activity have been developed. These models generally feature knockout of the *GBA1* gene (Xu et al., 2003; Enquist et al., 2007; Sardi et al., 2011), treatment with the GCase inhibitor conduritol β-epoxide (CBE; Manning-Boğ et al., 2009; Rocha et al., 2015) or mutations in the *GBA1* gene (Liu et al., 1998; Xu et al., 2003; Sardi et al., 2011; Cullen et al., 2011; Ginns et al., 2014). Even though most of these models exhibit dramatic reductions in GCase activity, many do not display nigrostriatal degeneration or PD-related motor symptoms (Farfel-Becker et al., 2019; Migdalska-Richards et al., 2017; Xu et al., 2003, 2011; Polinski et al., 2021; Sardi et al., 2011; Rocha et al., 2015). Importantly, the presence of a mutation in the *GBA1* gene is not uniformly causal for developing PD. This is evidenced through the vast majority of *GBA1* mutation carriers that do not develop PD, indicating a likely involvement for other factors working synergistically with *GBA1* mutations to lead to PD-related pathology and impairments. As such, models involving a second pathological insult have been developed to examine the relationship between *GBA1* mutations and other drivers of pathology.

Overexpression of aSyn is often chosen as the second pathological insult due to the demonstrated relationship between aSyn and GCase. Specifically, total and oligomeric forms of aSyn have been found to be elevated in the brain of patients carrying *GBA1* mutations (Mazzulli et al., 2011), the GCase protein has been found to be a component of aSyn-positive pathological inclusions in the brain of *GBA1* mutation-positive PD patients (Goker-Alpan et al., 2010), direct associations between aSyn and GCase have been demonstrated (Yap et al., 2011), and GCase levels and activity are demonstrably lower in brain tissue of sporadic PD patients with aSyn pathology as compared to controls (Mazzulli et al., 2011; Murphy and Halliday, 2014). Furthermore, a bidirectional loop has been proposed for the relationship with aSyn and GCase. Decreased GCase activity can lead to increased aSyn levels and aggregation through impairment of lysosomal function and accumulation of GCase substrates that promote aSyn aggregation (Manning-Boğ et al., 2009; Pitcairn et al., 2019; Mazzulli et al., 2011; Taguchi et al., 2017; Bae et al., 2015). Aggregation of aSyn, either through GCase deficiency-related mechanisms or GCase-independent mechanisms, can reduce GCase activity by impairing intracellular trafficking to prevent GCase localization to the lysosome and lysosomal maturation (Mazzulli et al., 2011; Gegg et al., 2012).

Various murine models have been developed to explore the relationship between aSyn overexpression and mutations in the *GBA1* gene. In 2014, Fishbein et al. crossed a heterozygous *GBA1* L444P mouse with a model overexpressing wild-type (WT) or A53T mutant human aSyn. These double transgenic lines displayed a further decrease in GCase protein and activity as compared to the single transgenic lines (Fishbein et al., 2014). In addition, the motor phenotypes in the aSyn overexpression lines were exacerbated by the *GBA1* L444P mutation. With regard to aSyn expression and pathology, however, results were method/region dependent as immunostaining showed an increase in total and phosphorylated Ser129 (pS129) aSyn levels in the hippocampus of the double transgenic line, whereas western blotting of whole brain homogenate showed no change (Fishbein et al., 2014). Similarly, Taguchi et al. (2017) generated *GBA1* L444P and N370S lines and crossed these lines with a mouse model overexpressing the A30P mutant human aSyn. In this case, the relationship between *GBA1* mutations and aSyn was more subtle, with no worsening of the motor defects and pS129 aSyn pathology being present only in the line carrying the human A30P aSyn plus heterozygous *GBA1* L444P mutation and knockout of the other *GBA1* allele (Taguchi et al., 2017). Migdalska-Richards et al. (2017) reported similar findings through the overexpression of human aSyn in the heterozygous *GBA1* L444P mouse using an aSyn-expressing viral vector. Although the authors' reported loss of dopaminergic neurons was more severe in the *GBA1* L444P model as compared to WT mice, pS129 aSyn pathology and dopaminergic signaling were not exacerbated by the *GBA1* mutation (Migdalska-Richards et al., 2017). Kim et al. in 2018 crossed a *GBA1* D409H model with an A53T human aSyn overexpression mouse. In this double transgenic line, the presence of aSyn overexpression further exacerbated the reduction of GCase activity in the midbrain, led to greater accumulation of the GCase substrate glucosylceramide (GlcCer) and increased levels of total aSyn. The double transgenic model also displayed nigrostriatal degeneration, motor deficits, aSyn aggregation and inflammation in the substantia nigra pars compacta (SNpc; Kim et al., 2018). Finally, Migdalska-Richards et al. (2020) injected aSyn preformed fibrils (PFFs) into the striatum of WT and heterozygous *GBA1* L444P mice and saw slight increases in pS129 aSyn pathology at 4 months post-injection in the *GBA1* L444P mice compared to WT mice. Taken together, these models illustrate a pathological relationship between aSyn and GCase, but to differing degrees depending on the model.

To investigate the impact of aSyn overexpression in a *GBA1* D409V knock-in (KI) mouse model, we pursued two approaches in this study. First, we generated a new model expressing the homozygous *GBA1* D409V mutation in the presence of WT human aSyn overexpression. Second, we injected aSyn PFFs into the striatum of the homozygous *GBA1* D409V KI model to seed pathology in the endogenous murine aSyn protein. These strategies were pursued to determine whether the *GBA1* D409V mutation worsened aSyn pathology and nigrostriatal degeneration in a constitutive aSyn overexpression model or a more aggressive, inducible model of aSyn pathology and nigrostriatal degeneration. The results of these studies are reported herein.

## RESULTS

### Generation of a novel mouse model combining decreased GCase activity with human WT aSyn overexpression

Within this study, we generated a new mouse model by crossing a line expressing the *GBA1* point mutation D409V knocked into the mouse *Gba1* locus (the *GBA1* D409V KI mouse) with a line overexpressing human wild-type aSyn (the mThy1-hSNCA mouse). Both lines have been characterized previously. The homozygous *GBA1* D409V KI mouse displays a drastic decrease in GCase activity with an increase in glycosphingolipid (GSL) substrates GlcCer and glucosylsphingosine (GlcSph) (Polinski et al., 2021). Regarding PD-related pathology, this line has an increase in dopamine turnover at 12 months of age without behavioral deficits, loss of dopaminergic neurons in the SNpc, robust aSyn pathology or neuroinflammation (Polinski et al., 2021). The mThy1-hSNCA mouse has stable overexpression of human WT aSyn in neurons and microglial activation at 10+ months of age, but no dopaminergic neuron loss in the SNpc (Choi et al., 2020). The new model resulting from the breeding of these two lines – denoted as the ‘*GBA1* D409V KI×mThy1-hSNCA’ mouse – is viable with no noticeable differences in breeding, litter size, weaning, development or sexual maturity.

### The *GBA1* D409V KI mutation increases aSyn pathology on a human aSyn overexpression background

**Fig. 1.**
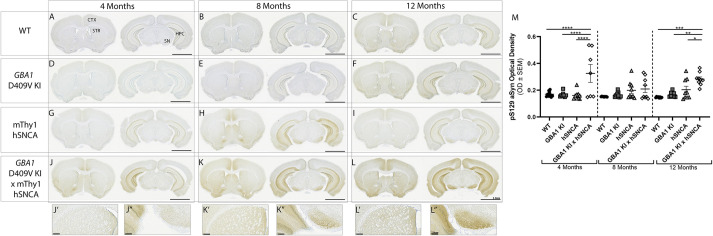
**Phosphorylated alpha-synuclein is increased in the *GBA1* D409V KI×mThy1-hSNCA mouse.** (A-L″) Representative images of immunohistochemical staining for pS129 aSyn in WT (A-C), *GBA1* D409V KI (D-F), mThy1-hSNCA (G-I) and *GBA1* D409V KI×mThy1-hSNCA (J-L) mice at 4, 8 and 12 months of age (*n*=9/group). WT mice (A-C) and *GBA1* D409V KI mice (D-F) displayed negligible levels of pS129 aSyn at all ages. mThy1-hSNCA mice (G-I) displayed variable, slight pS129 aSyn pathology in structures such as the hippocampus and substantia nigra at 8 months. (J-L″) *GBA1* D409V KI×mThy1-hSNCA mice displayed pS129 aSyn pathology in structures like the striatum (STR), cortex (CTX), hippocampus (HPC) and substantia nigra (SN) that accumulated with age. Primed images (J′,K′,L′) are higher magnification images of the striatum and double-primed images (J″,K″,L″) are higher magnification images of the SN. Scale bars: 2.5 mm (A-I,J,K,L), 224 μm (J′,J″,K′,K″,L′,L″). (M) Densitometric area quantification of positive pS129 aSyn signal in the SN confirmed an increase in pS129 aSyn in *GBA1* D409V KI×mThy1-hSNCA mice at 4 and 12 months of age. Variability at 8 months of age was observed in pS129 aSyn in mThy1-hSNCA and *GBA1* D409 V KI×mThy1-hSNCA mice. Significant differences from a two-way ANOVA with Tukey post hoc tests are reported as follows: *P<0.05; **P<0.01; ***P<0.001; ****P<0.0001.

We first analyzed the presence of pS129 aSyn in WT, mThy1-hSNCA, *GBA1* D409V KI and *GBA1* D409V KI×mThy1-hSNCA mice using immunohistochemistry. Qualitatively, the increases in aSyn pathology in the *GBA1* D409V KI×mThy1-hSNCA mouse were the most striking in the hippocampus and substantia nigra (SN), with more subtle increases in pS129 aSyn in the cortex and striatum. Quantification of the staining intensity in the SN revealed significant differences across genotypes [Fig. 1M; *F*(3, 91)=17.64, *P*<0.0001]. Specifically, *GBA1* D409V KI×mThy1-hSNCA had significantly higher pS129 aSyn than the other genotypes at 4 and 12 months of age. While variability in pS129 aSyn in *GBA1* D409V KI×mThy1-hSNCA mice was high at 4 months of age, pS129 aSyn was consistently elevated in *GBA1* D409V KI×mThy1-hSNCA at 12 months of age as compared to WT mice (*P*=0.0001) and mice with the single *GBA1* D409V (*P*=0.0013) or mThy1-hSNCA (*P*=0.0450) mutations (Fig. 1).

### Neuroinflammation is not exacerbated by the *GBA1* D409V mutation in mThy1-hSNCA mice

Central and peripheral inflammation is a common pathological signature of PD (Tan et al., 2020), with increasing evidence that *GBA1* mutations can further exacerbate inflammation (Chahine et al., 2013; Dzamko et al., 2015). To determine whether neuroinflammation was present in the nigrostriatal system when the *GBA1* D409V mutation was combined with the mThy1-hSNCA mutation, we used immunostaining for microglia and astrocytes in the SN of *GBA1* D409V KI×mThy1-hSNCA mice at 4, 8 and 12 months of age (Fig. S1). Staining intensity quantification revealed an effect of genotype on microglial activation and astrocyte reactivity (two-way ANOVA results reported in Table S1). However, no elevation in microglia (Fig. S1M) or astrocyte reactivity (Fig. S1N) was observed at any age in *GBA1* D409V KI×mThy1-hSNCA mice besides a transient increase in astrocyte reactivity at 8 months of age as compared to WT mice (*P*=0.0094). Rather, microglia activation was lower in *GBA1* D409V KI and *GBA1* D409V KI×mThy1-hSNCA mice as compared to mThy1-hSNCA mice at 12 months of age (*P*=0.0005 and *P*<0.0001, respectively). A similar pattern was observed in astrocyte reactivity at 12 months of age, with lower levels in *GBA1* D409V KI×mThy1-hSNCA versus mThy1-hSNCA mice (*P*=0.0232) and *GBA1* D409V KI versus WT (*P*=0.0279) and mThy1-hSNCA (*P*=0.0053) mice.

### The *GBA1* D409V KI mutation affects motor function in an aSyn transgenic mouse

To understand the gross impact of decreased GCase enzyme activity on PD-related phenotypes in an aSyn-overexpressing mouse model, behavioral readouts of motor impairment were assessed. Cohorts of C57Bl/6 WT mice, *GBA1* D409V KI, mThy1-hSNCA mice and *GBA1* D409V KI×mThy1-hSNCA mice were generated and aged to 4, 8 and 12 months. At these timepoints, mice underwent testing to assess autonomic function, neuromuscular function, sensorimotor function, central nervous system (CNS) excitability, CNS activity and physiology.

**Fig. 2.**
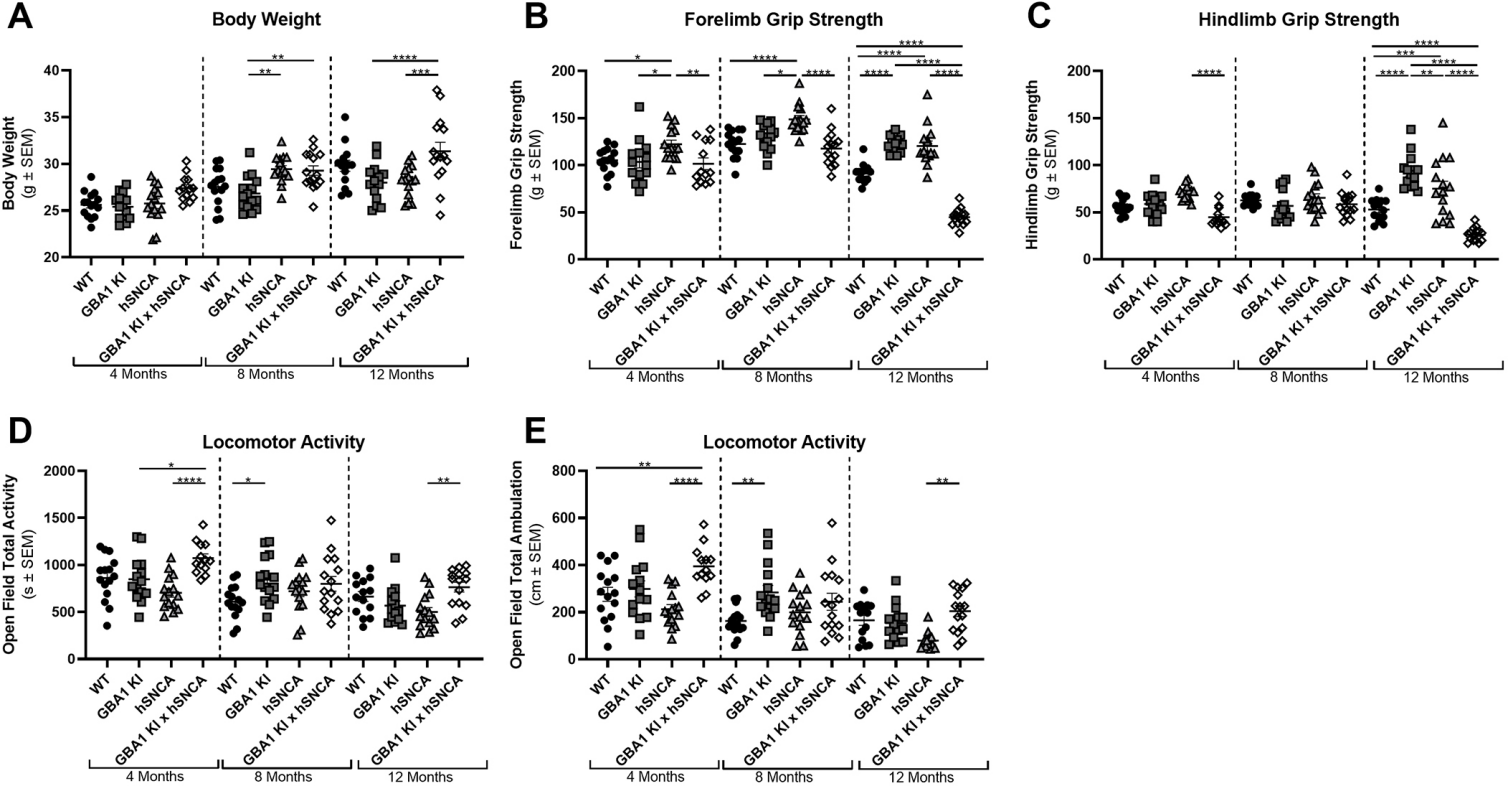
**The *GBA1* D409V KI×mThy1-hSNCA mouse displays decreased grip strength at 12 months of age.** Physiological and behavioral assessments of WT, *GBA1* D409V KI, mThy1-hSNCA and *GBA1* D409V KI×mThy1-hSNCA mice at 4, 8 and 12 months of age (*n*=13-15/group). (A) Assessment of body weight (in g) indicated roughly equivalent weight between genotypes. (B) Forelimb grip strength (in g) was increased at all ages in mThy1-hSNCA mice and decreased in *GBA1* D409V KI×mThy1-hSNCA mice at 12 months of age. (C) Hindlimb grip strength (in g) was increased in *GBA1* D409V KI and mThy1-hSNCA mice at 12 months and decreased in *GBA1* D409V KI×mThy1-hSNCA mice at 12 months of age. (D) Total locomotor activity (in s) was relatively similar between genotypes, with some minor differences between groups that did not persist across all timepoints. (E) Total ambulation (in cm) was relatively similar between groups with a slight increase in *GBA1* D409V KI×mThy1-hSNCA mice as compared to WT and mThy1-hSNCA controls at 4 months of age only. Significant differences from a two-way ANOVA with Tukey post hoc tests are reported as follows: *P<0.05; **P<0.01; ***P<0.001; ****P<0.0001.

In the evaluation of physiology, it was noticed that body weight differed between lines at mid/late timepoints (Fig. 2A), but these differences varied between genotypes and timepoints (two-way ANOVA results reported in Table S1). Within the 8 month cohort, mThy1-hSNCA mice generally weighed more than *GBA1* D409V KI mice (difference of 2.6±0.74 g, indicated as mean±s.e.m), as did *GBA1* D409V KI×mThy1-hSNCA mice (difference of 2.4±0.74 g). The increase in mThy1-hSNCA body weight did not persist in the 12 month cohort. In the 12 month cohort, however, *GBA1* D409V KI×mThy1-hSNCA mice were significantly larger than mThy1-hSNCA mice (difference of 3.2±0.74 g) and *GBA1* D409V KI mice (difference of 3.4±0.75 g), although the increase in weight in *GBA1* D409V KI×mThy1-hSNCA mice was quite variable and not significant as compared to WT mice. The fluctuations between the different aged cohorts corresponded to a significant effect of age on body weight.

In the analysis of motor phenotypes, multiple differences were observed between genotypes. Forelimb grip strength was consistently different between genotypes in the various age cohorts (Fig. 2B; two-way ANOVA and post-hoc test results reported in Table S1), with an interesting elevation in forelimb grip strength in mThy1-hSNCA mice at all ages and drop in forelimb grip strength in *GBA1* D409V KI×mThy1-hSNCA mice at 12 months of age. Specifically, mThy1-hSNCA mice displayed a significant increase in forelimb grip strength as compared to WT and *GBA1* D409V KI×mThy1-hSNCA at all ages. mThy1-hSNCA mice also displayed greater grip strength as compared to *GBA1* D409V KI mice at 4 and 8 months of age, but not at the final 12 month timepoint. At 12 months of age, *GBA1* D409V KI mice displayed an increase in grip strength as compared to WT mice as well. Although comparable to WT and *GBA1* D409V KI mice at 4 and 8 months of age, at 12 months of age, the *GBA1* D409V KI×Thy1-hSNCA mice displayed lower grip strength, which resulted in significant differences with WT and *GBA1* D409V KI mice. Similar patterns were observed for hindlimb grip strength (Fig. 2C), particularly at 12 months of age where *GBA1* D409V KI and mThy1-hSNCA mice had increased strength compared to WT, whereas hindlimb grip strength was significantly reduced in *GBA1* D409V KI×mThy1-hSNCA mice as compared to all other genotypes.

Locomotor activity (Fig. 2D) and total ambulation (Fig. 2E) measures also uncovered differences between genotypes, but these differences were not consistent or striking (two-way ANOVA statistical test results reported in Table S1). In both readouts, *GBA1* D409V KI×mThy1-hSNCA mice displayed elevated measures at 4 months of age as compared to some, but not all, controls. Specifically, *GBA1* D409V KI×mThy1-hSNCA mice showed increased activity as compared to *GBA1* D409V KI and mThy1-hSNCA mice, with a trend towards an increase compared to WT mice (*P=*0.0518). Ambulation was increased at 4 months in the *GBA1* D409V KI×mThy1-hSNCA mice as compared to WT and mThy1-hSNCA mice, with only a trend towards increase compared to *GBA1* D409V KI mice (*P=*0.0517). These increases were not sustained at 8 months and at 12 months, as the only significant increase was measured against mThy1-hSNCA mice. Interestingly, the *GBA1* D409V KI mice showed an increase in activity and ambulation as compared to WT, but only transiently at 8 months. Some of these differences could be due to age-related changes in open-field activity and ambulation. For instance, WT and *GBA1* D409V KI×mThy1-hSNCA mice displayed a significant reduction in total activity and ambulation at 8 months as compared to 4 months, which could help explain the transient increase in *GBA1* D409V KI mice at 8 months. Furthermore, the mThy1-hSNCA mice displayed a significant reduction in total activity and ambulation between 8 and 12 months, leading to the differences observed between this model and the *GBA1* D409V KI×mThy1-hSNCA at the 12 month timepoint.

### *GBA1* D409V KI mutation affects nigrostriatal function in aSyn transgenic mice

Dysregulated dopamine signaling in the striatum is a sign of dopaminergic neuron dysfunction in the SNpc and a hallmark of PD-causing stereotypical motor deficits (Sossi et al., 2004). Using a previously described high-performance liquid chromatography with tandem mass spectrometry (HPLC/MS/MS) method (Polinski et al., 2021), we assessed dopamine (DA), the dopamine metabolites 3,4-dihydroxyphenylacetic acid (DOPAC) and homovanillic acid (HVA), serotonin (5-HT) and the serotonin metabolite 5-hydroxyindole-3-acetic acid (5-HIAA) in WT, *GBA1* D409V KI, mThy1-hSNCA and *GBA1* D409V KI×mThy1-hSNCA mice at 4, 8 and 12 months of age.

**Fig. 3.**
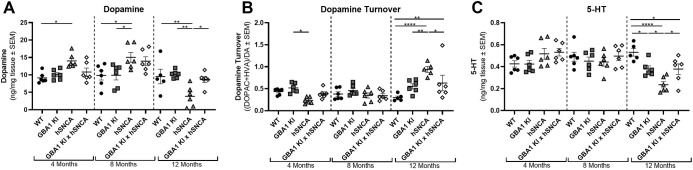
**The *GBA1* D409V KI×mThy1-hSNCA mouse displays increased striatal dopamine turnover and decreased serotonin at 12 months of age.** (A-C) Striatal dopamine (A), dopamine turnover (B) and serotonin (5-HT) (C) were measured in WT, *GBA1* D409V KI, mThy1-hSNCA and *GBA1* D409V KI×mThy1-hSNCA mice at 4, 8 and 12 months of age (*n*=6/group). (A) Dopamine levels were unchanged in the *GBA1* D409V KI×mThy1-hSNCA mouse at all timepoints, with mThy1-hSNCA mice showing an increase at 4 and 8 months followed by a decrease at 12 months as compared to WT controls. (B) Dopamine turnover was increased at 12 months of age in mThy1-hSNCA mice, and to a lesser extent in *GBA1* D409V KI×mThy1-hSNCA mice. (C) Serotonin levels were significantly decreased in mThy1-hSNCA mice, and to a lesser extent in *GBA1* D409V KI and *GBA1* D409V KI×mThy1-hSNCA mice at 12 months of age. Significant differences from a two-way ANOVA with Tukey post hoc tests are reported as follows: *P<0.05; **P<0.01; ****P<0.0001. *GBA1* D409V versus WT control data are also reported in Polinski et al. (2021).

On a holistic level, dopamine levels did not differ between genotypes, but age-related differences were observed (Fig. 3A; two-way ANOVA results reported in Table S1). In particular, the mThy1-hSNCA mice displayed higher dopamine levels than WT mice at 4 and 8 months, and higher dopamine levels than *GBA1* D409V KI mice at 8 months; however, a precipitous drop in dopamine levels in this model at 12 months of age led to significantly lower readouts in mThy1-hSNCA mice as compared to all genotypes at 12 months of age. Interestingly, the *GBA1* D409V KI×mThy1-hSNCA mice displayed normal dopamine levels at all ages.

Similarly, dopamine turnover levels only trended towards a genotype difference, but demonstrated age-related changes (Fig. 3B; two-way ANOVA results reported in Table S1). Age-related changes were mostly driven by increases at 12 months in the mThy1-hSNCA mice. The *GBA1* D409V KI×mThy1-hSNCA line also displayed an increase in dopamine turnover compared to WT mice at 12 months, but this effect was more limited and mostly driven by high turnover in one animal. Interestingly, there was no significant difference in dopamine turnover at 12 months when comparing WT to *GBA1* D409V KI mice (*P=*0.0689), even though this difference was statistically significant when analyzing the same dataset in a different study (Polinski et al., 2021). This is due to the small magnitude of the difference.

Altered dopamine turnover was related to changes observed in DOPAC (Fig. S2A) and HVA levels (Fig. S2B). Specifically, lower dopamine turnover in mThy1-hSNCA versus *GBA1* D409V KI mice at 4 months of age (Fig. 3B) was due to differences in DOPAC between these groups (*P=*0.0104; Fig. S2A) . Differences in HVA between genotypes was also observed (Fig. S2B), but the increase in HVA levels in mThy1-hSNCA versus WT and *GBA1* D409V KI×mThy1-hSNCA versus WT was not large enough to cause significant changes in overall dopamine turnover (Fig. 3B). At 12 months of age, *GBA1* D409V KI mice displayed increased DOPAC levels as compared to WT and mThy1-hSNCA mice. HVA levels, on the other hand, were increased in the *GBA1* D409V KI×mThy1-hSNCA mice as compared to WT and mThy1-hSNCA mice. Interestingly, there was no main effect of age on DOPAC or HVA levels, although an age-related effect was seen in dopamine levels and dopamine turnover (two-way ANOVA statistical test results reported in Table S1).

Serotonin and serotonin metabolites were also assessed in the striatum as a readout of striatal cholinergic inputs regulating motor function and mood (Beaudoin-Gobert and Sgambato-Faure, 2014). Interestingly, striatal serotonin levels decreased in all transgenic animals as compared to WT (Fig. 3C; two-way ANOVA statistical test results reported in Table S1). The decrease was greatest in mThy1-hSNCA mice compared to WT mice, with lesser decreases in *GBA1* D409V KI×mThy1-hSNCA and *GBA1* D409V KI mice. The decrease in serotonin levels in mThy1-hSNCA mice also led to significant differences between this line and *GBA1* D409V KI and *GBA1* D409V KI×mThy1-hSNCA mice. These differences were mostly driven by an age-related decrease in the mThy1-hSNCA mice. The serotonin metabolite 5-HIAA also differed by genotype and age (Fig. S2C; two-way ANOVA test results reported in Table S1). The genotype differences were driven by differences in mThy1-hSNCA versus *GBA1* D409V KI×mThy1-hSNCA mice at 8 months and *GBA1* D409V KI mice at 12 months.

**Fig. 4.**
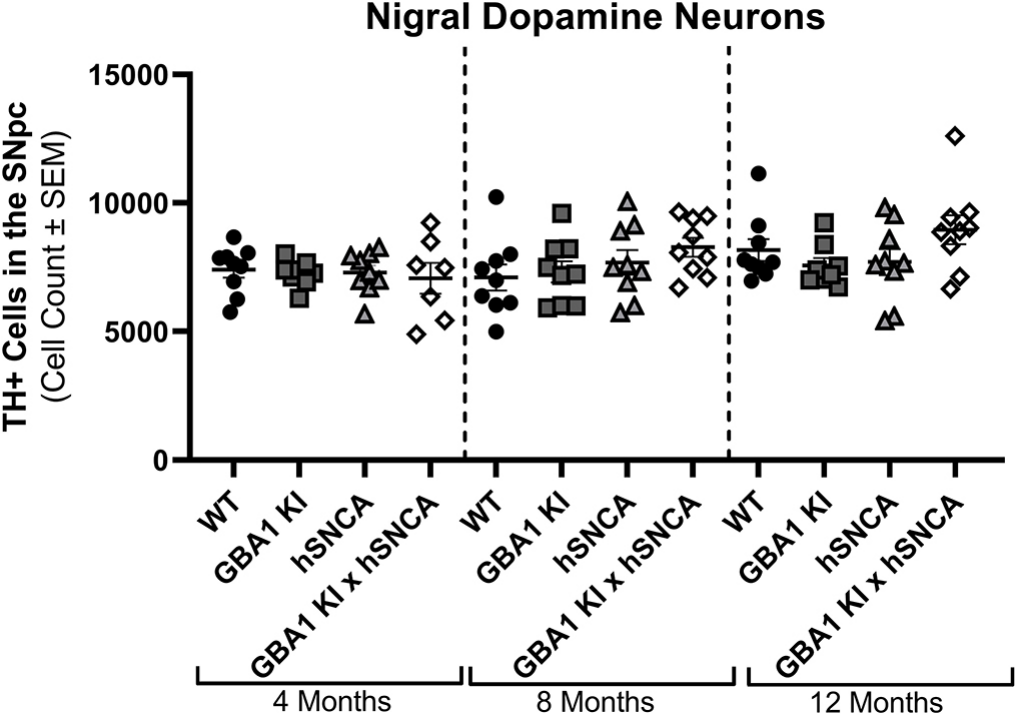
**Nigral dopamine neurons are preserved in the *GBA1* D409V KI×mThy1-hSNCA mouse.** Stereological estimates of dopaminergic neurons in the SNpc as denoted by tyrosine hydroxylase immunoreactivity (TH^+^) revealed no differences between any genotypes at any age (*n*=9/group). No significant differences were identified from a two-way ANOVA. *GBA1* D409V versus WT control data are also reported in Polinski et al. (2021); mThy1-hSNCA and WT control data are also reported in Choi et al. (2020).

Importantly, the changes in neurochemistry were not due to frank loss of dopaminergic neurons in the SNpc, as no genotype-related differences were observed using unbiased stereology [*F*(3, 92)=1.522, *P=*0.2140; Fig. 4; Choi et al., 2020; Polinski et al., 2021].

### Development of an inducible model of aSyn pathology in the context of the *GBA1* D409V KI mutation

**Fig. 5.**
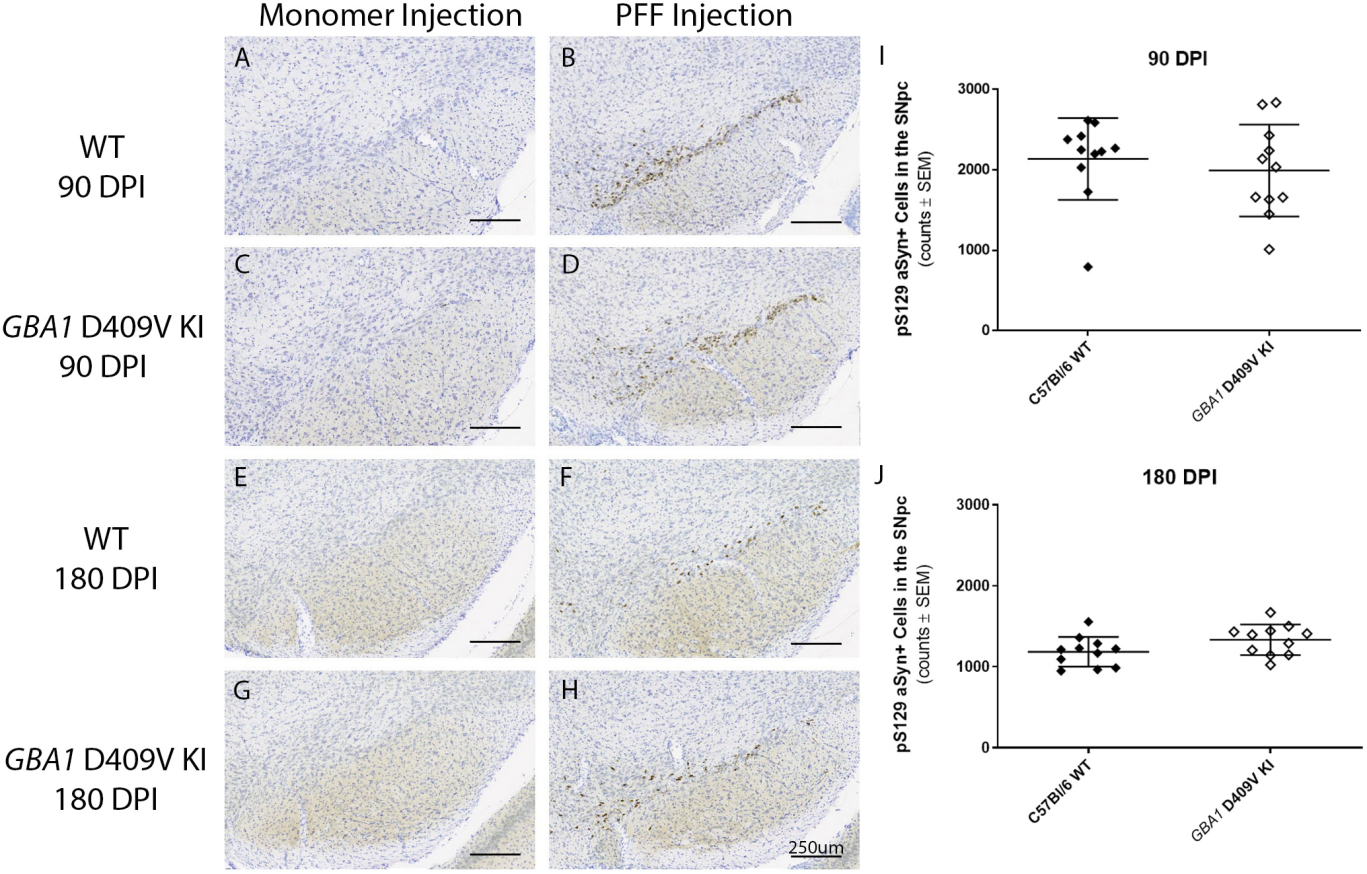
**Injection of alpha-synuclein preformed fibrils leads to similar increases in phosphorylated alpha-synuclein in both wild-type and *GBA1* D409V KI mice.** (A-H) Representative images of immunohistochemical staining for pS129 aSyn in WT and *GBA1* D409V KI mice following aSyn monomer (A,E and C,G, respectively) or PFF (B,F and D,H, respectively) injection (*n*=11/group). Injection of aSyn PFFs led to robust pS129 aSyn staining in the SN at 90 DPI (B,D) and to a lesser extent at 180 DPI (F,H). Injection of aSyn monomer did not lead to pS129 aSyn-immunoreactive cells at either timepoint (A,C,E,G). Scale bars: 250 μm. (I-J) Stereological quantitation of pS129 aSyn-immunoreactive cells in the SN of aSyn PFF-injected mice revealed no differences between genotype in the number of pS129 aSyn-positive cells at 90 (I) or 180 (J) DPI. No significant differences were identified by unpaired two-tailed *t*-test.

In order to assess whether developmental compensation or lack of substantial aSyn expression/pathology in the mThy1-hSNCA mouse resulted in the lack of nigrostriatal degeneration in the presence of exacerbated aSyn pathology in the *GBA1* D409V KI×mThy1-hSNCA line, we next decided to combine the *GBA1* D409V mutation with a more pronounced PD model that demonstrates synuclein pathology and nigrostriatal dysfunction – the aSyn PFF model. We injected either monomeric mouse aSyn or mouse aSyn PFFs in the striatum of WT and *GBA1* D409V KI mice and examined end points at 90 and 180 days post-injection (DPI) to assess nigrostriatal integrity. To confirm successful PFF formation and injection, we assessed pS129 aSyn pathology. As has been previously reported, intrastriatal injection of aSyn PFFs – but not aSyn monomers – led to the development of pS129 aSyn pathology (Fig. 5).

### The *GBA1* D409V KI mutation does not meaningfully increase aSyn pathology or neuroinflammation in the aSyn PFF model

To determine whether the *GBA1* D409V mutation exacerbated synuclein pathology driven by aSyn PFF injection, pS129 aSyn staining was performed at 90 and 180 DPI, with stereological estimates of pS129 aSyn-positive cells performed in the ipsilateral SNpc of PFF-injected animals (no pS129 aSyn signal was detected in monomer-injected animals; Fig. 5). The *GBA1* D409V mutation did not exacerbate aSyn pathology at 90 days following aSyn PFF injection (*P=*0.5383). Although a slight trend towards more pS129 aSyn-positive cells in the SNpc of *GBA1* D409V KI versus WT mice did appear at 180 DPI, this difference was minor and not statistically significant (*P=*0.0756).

Neuroinflammation was also assessed in the SNpc to determine whether the *GBA1* D409V mutation in the context of the pathology induced by aSyn PFF-injections resulted in an inflammatory response (Fig. S3). No significant differences in reactive microglia or astrocytes were observed at 90 or 180 DPI in the *GBA1* D409V KI versus WT mice (statistical test results reported in Table S2).

### The *GBA1* D409V KI mutation does not exacerbate nigrostriatal pathology in the aSyn PFF model

**Fig. 6.**
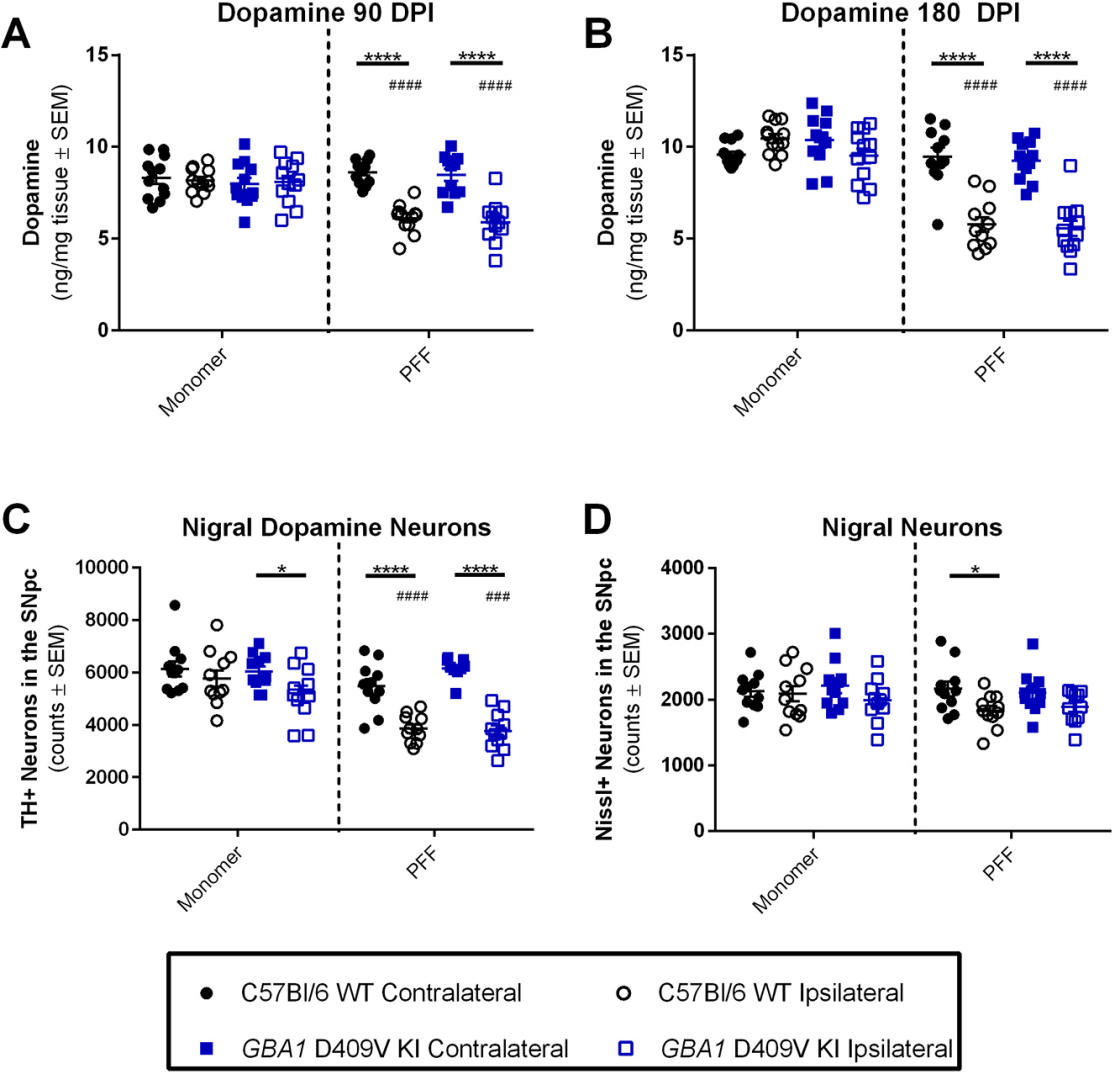
**The *GBA1* D409V mutation does not exacerbate nigrostriatal degeneration induced by alpha-synuclein preformed fibrils.** (A,B) Striatal dopamine was significantly decreased in the aSyn PFF-injected hemisphere as compared to the contralateral hemisphere and monomer-injected animals (*n*=11-12/group) at 90 (A) and 180 (B) DPI. (C) Stereological estimates of dopaminergic neurons in the SNpc as denoted by tyrosine hydroxylase immunoreactivity (TH^+^) revealed significant effects of aSyn PFF injection but not genotype at 180 DPI (*n*=11/group). (D) Stereological estimates of neurons in the SNpc as denoted by Nissl immunoreactivity (Nissl^+^) revealed a slight decrease in neuron numbers in the ipsilateral versus contralateral hemisphere of WT mice injected with aSyn PFFs at 180 DPI (*n*=11/group). Significant differences between injectate are represented as follows: ^###^*P*<0.001, ^####^*P*<0.0001. Significant differences between hemisphere from a repeated measures two-way ANOVA with Sidak post hoc tests are represented as follows: *P<0.05; ****P<0.0001. For all analyses, no genotype differences within injectate/hemisphere groups were observed.

After confirming successful PFF formation and injection, we assessed nigrostriatal pathology through striatal neurochemical analysis and neuronal cell counts in the SNpc. As has been observed previously, injection of aSyn PFFs decreased striatal dopamine levels at 90 DPI (Fig. 6A) and 180 DPI (Fig. 6B; repeated measures two-way ANOVA test results reported in Table S2). At both timepoints, injection of aSyn PFFs induced a substantial decrease in dopamine levels as compared to injection of monomers (*P*<0.0001), and this decrease was isolated to the injected hemisphere (*P*<0.0001). No differences were observed between genotypes.

At 90 DPI, dopamine turnover was significantly affected by the genotype, but no effects of the injectate or hemisphere were observed (Fig. S4E; repeated measures two-way ANOVA test results reported in Table S2). Interestingly, dopamine turnover was significantly increased in *GBA1* D409V KI mice when comparing the injected hemisphere (regardless of the injectate) and uninjected hemisphere following aSyn PFF administration. This effect was not observed at 180 DPI, however (Fig. S4F; repeated measures two-way ANOVA results reported in Table S2). At 180 DPI, the only genotype-related difference was in the uninjected hemisphere following aSyn monomer administration, and, in this case, the *GBA1* D409V KI mice displayed lower dopamine turnover as compared to WT controls. Whereas no injectate-related differences were reported at 90 DPI, at 180 DPI, a clear effect of PFF injection was observed in *GBA1* D409V KI mice; dopamine turnover was increased following PFF injection when compared to monomer injection in both the ipsilateral (*P*<0.0001) and contralateral (*P=*0.0117) hemisphere, with significant differences between the ipsilateral and contralateral PFF-injected hemispheres as well (*P=*0.0442).

Similar to dopamine levels, DOPAC levels were decreased following PFF injection at 90 DPI and 180 DPI (Fig. S4A,B; repeated measures two-way ANOVA test results reported in Table S2). At both timepoints, injection of aSyn PFFs induced a decrease in DOPAC levels as compared to injection of monomers, and this decrease was isolated to the injected hemisphere. No differences were observed between genotypes (*P*>0.05). The levels of HVA, another dopamine metabolite, were also measured, but appeared to have more variation than DOPAC levels. At 90 and 180 DPI, HVA levels were significantly different between groups (Fig. S4C,D; repeated measures two-way ANOVA results reported in Table S2). At 90 DPI, HVA levels were significantly increased in *GBA1* D409V KI versus WT mice when comparing the injected hemisphere (regardless of injectate) and uninjected hemisphere following aSyn PFF administration. These results mirror the dopamine turnover differences (Fig. S4E) and likely drive the effect. These differences did not persist at the 180 DPI timepoint (Fig. S4C,D). Differences within genotypes related to the injections were quite variable and inconsistent between timepoints.

Serotonin and the serotonin metabolite 5-HIAA levels were also measured. Differences in serotonin levels were only observed at 90 DPI (Fig. S4G) and were primarily driven by an increase in serotonin in the PFF-injected hemisphere of the *GBA1* D409V KI mice (repeated measures two-way ANOVA test results reported in Table S2). No differences were observed between genotype, injection or injectate at 180 DPI (Fig. S4H). Similarly, changes in 5-HIAA levels were primarily observed at 90 DPI (Fig. S4I). Here, genotype-related differences were observed following aSyn PFF injection in both the contralateral and ipsilateral hemispheres, with levels being slightly higher in *GBA1* D409V KI mice. This difference did not persist at 180 DPI (Fig. S4J). Hemisphere differences in 5-HIAA levels were observed variably and inconsistently, possibly driven by small group sizes rather than a robust effect of injection.

Finally, to determine whether the striatal dopaminergic signaling alterations were due to a loss of dopaminergic cells in the SNpc, unbiased stereology was performed at 180 DPI (Fig. 6C-D). Injection of aSyn PFFs resulted in decreased tyrosine hydroxylase (TH)-immunoreactive neurons in the SNpc (Fig. 6C; repeated measures two-way ANOVA test results reported in Table S2). A decrease in TH-immunoreactive neurons was shown in the injected hemisphere following aSyn PFF administration, leading to significant differences between injectate (monomer versus PFF) and hemisphere (ipsilateral versus contralateral). No differences were observed between genotypes. To determine whether this was due to downregulation of TH expression or frank cell loss, unbiased stereology was also performed on Nissl-immunoreactive cells. For Nissl stereology, no differences were observed between monomer versus PFF injection or for the genotype (Fig. 6D; repeated measures two-way ANOVA test results reported in Table S2). There was only a slight difference in WT mice following PFF injection when comparing the ipsilateral versus the contralateral hemisphere, with a similar non-significant trend in the *GBA1* D409V KI mice. Taken together, these results generally replicate previous observations of the effect of aSyn PFFs on the nigrostriatal system as it pertains to dopaminergic signaling and dopaminergic neuron integrity in the SNpc. However, with regard to whether the *GBA1* D409V KI mutation exacerbates PD-related nigrostriatal dysfunction, the mutation does not appear to have an effect.

### aSyn PFF treatment does not affect GCase activity

**Fig. 7.**
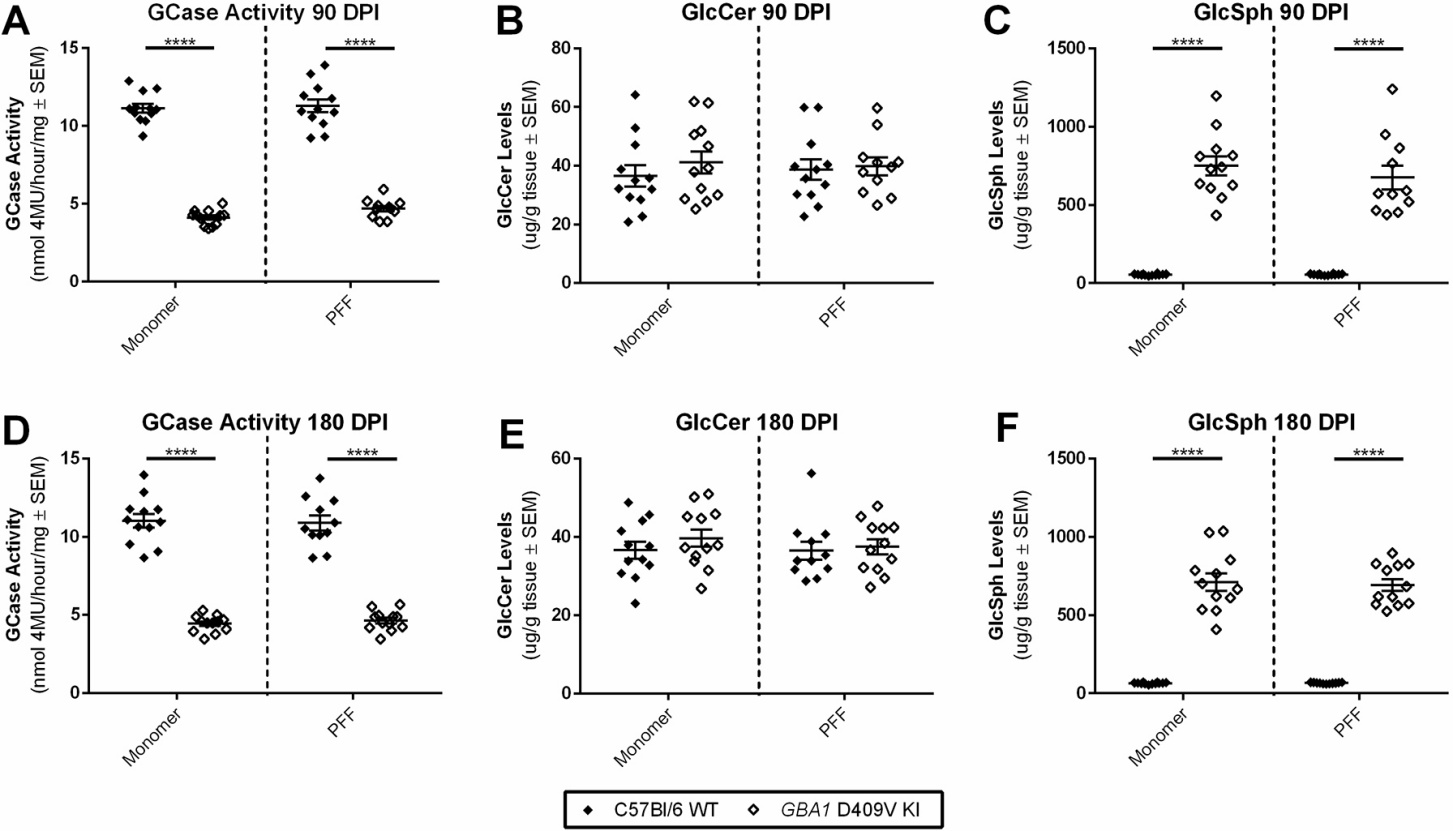
**Alpha-synuclein preformed fibril injection does not exacerbate the decrease in GCase activity or substrate accumulation.** (A-F) GCase (A,D), GlcCer (B,E) and GlcSph (D,F) levels were measured in brain homogenates of WT and *GBA1* D409V KI mice at 90 (A-C) and 180 (D-F) DPI of aSyn monomer or PFFs (*n*=11/age). (A,D) GCase levels as assessed by the CBE/4-MUG method were significantly decreased in the *GBA1* D409V KI mice as compared to WT controls at 90 DPI (A) and 180 DPI (D), but the decrease was not exacerbated by aSyn PFF administration. (B,E) GlcCer levels were unchanged in *GBA1* D409V KI mice as compared to WT mice at 90 DPI (B) and 180 DPI (E), regardless of aSyn PFF injection. (C,F) GlcSph levels were significantly increased in *GBA1* D409V KI mice as compared to WT mice, but this effect was not affected by aSyn PFF treatment at 90 DPI (C) or 180 DPI (F). Significant differences from a two-way ANOVA with Sidak post hoc tests are represented as follows: ****P<0.0001.

As a feed-forward loop between increased aSyn and decreased GCase activity has been postulated (Pitcairn et al., 2019; Mazzulli et al., 2011; Taguchi et al., 2017; Bae et al., 2015; Sardi et al., 2011), we also wanted to assess whether injection of aSyn PFFs and the resulting synuclein pathology led to further changes in GCase activity or substrate accumulation in the *GBA1* D409V KI mouse model. To do this, we employed the CBE/4-methylumbelliferyl-β-D-glucopyranoside (4-MUG) method using lysates from the injected hemisphere (two-way ANOVA test results reported in Table S2). As was shown previously (Polinski et al., 2021), the *GBA1* D409V KI mutation significantly decreases GCase activity (Fig. 7A,D) and increases the GCase substrate GlcSph (Fig. 7C,F) without affecting the GCase substrate GlcCer (Fig. 7B,E). However, GCase activity and substrate levels were not affected by PFF injection at either timepoint. Taken together, these results indicate that aSyn PFF injection in the *GBA1* D409V KI mouse does not exacerbate changes in GCase activity or substrate levels under these experimental paradigms.

## DISCUSSION

Although the exact cause of most cases of PD is unknown, multiple lines of evidence point to conserved mechanistic pathways in genetic and idiopathic cases. GCase and aSyn are a prime example of distinct targets converging on similar pathways to potentially cause or exacerbate PD-related neurodegeneration. Mutations in the *GBA1* gene are the most common genetic risk factor for idiopathic PD (Rosenbloom et al., 2011; Sidransky et al., 2009), and patients with *GBA1* mutations develop nigrostriatal degeneration and aSyn pathology similar to sporadic PD patients (Blandini et al., 2018; Goker-Alpan et al., 2010; Neumann et al., 2009). Reduced GCase activity has also been reported in idiopathic PD patients without mutations in the *GBA1* gene (Mazzulli et al., 2011; Gegg et al., 2012; Murphy and Halliday, 2014; Ortega et al., 2016; Atashrazm et al., 2018). Preclinically, a relationship between aSyn and GCase has been demonstrated and a feed-forward mechanism for pathology has been proposed, with increased aSyn leading to further decreases in GCase activity and decreased GCase activity, leading to further accumulation of aSyn (Bae et al., 2015; Gegg et al., 2012; Mazzulli et al., 2011).

To understand the relationship between aSyn and GCase in PD pathology, recent work has led to the development of two-hit models that (1) overexpress aSyn or exhibit aSyn pathology, and (2) demonstrate reduced GCase activity. In general, these models support the bidirectional nature of the GCase-aSyn relationship, though to varying degrees depending on the mechanism for aSyn overexpression, *SNCA* mutation or *GBA1* mutation (Fishbein et al., 2014; Taguchi et al., 2017; Migdalska-Richards et al., 2017; Kim et al., 2018; Migdalska-Richards et al., 2020). To determine whether the *GBA1* D409V mutation – which results in significant loss of GCase activity and accumulation of GSL substrates (Polinski et al., 2021; Clarke et al., 2019; Burbulla et al., 2019) – exacerbates aSyn-mediated pathology, we generated and characterized nigrostriatal pathology in two new models within this study.

In the first model, we crossed the *GBA1* D409V KI mouse (Polinski et al., 2021; Clarke et al., 2019, Burbulla et al., 2019) with a transgenic line overexpressing human wild-type aSyn under the Thy1 promoter (Choi et al., 2020) to create a new model with constitutive aSyn overexpression on a homozygous *GBA1* D409V background. This model exhibited increased aSyn pathology, especially at 12 months of age (Fig. 1). Despite this increase in pS129 aSyn at late ages, the *GBA1* D409V KI×mThy1-hSNCA mouse did not develop overt nigrostriatal degeneration (Figs 3 and 4). Of note, total aSyn protein expression was not measured in these animals. Therefore, we cannot discount the possibility that the *GBA1* D409V mutation affected the expression of aSyn in the mThy1-hSNCA mouse. Although this is a possibility, the *GBA1* D409V KI×mThy1-hSNCA mouse did display increased pS129 aSyn versus the relevant controls, and *GBA1* mutations have not been shown to decrease aSyn protein levels in other synuclein transgenic mouse lines (Fishbein et al., 2014; Kim et al., 2018). In fact, Kim et al. (2018) showed that the *GBA1* D409H mutation increased aSyn protein levels in an A53T aSyn transgenic mouse in a dose-dependent fashion. Given this, future studies should measure aSyn protein expression in this and other models investigating the convergence of GCase and aSyn.

The *GBA1* D409V KI×mThy1-hSNCA mice did, however, display a significant reduction in forelimb and hindlimb grip strength at 12 months of age (Fig. 2B,C). This finding is intriguing as it cannot be directly attributed to nigrostriatal pathology, as the magnitude of dopaminergic signaling changes are minimal (Fig. 3) and the loss of dopaminergic neurons in the SNpc was not observed (Fig. 4). Interestingly, decreased grip strength without nigrostriatal degeneration was recently reported in another dual-hit aSyn-*GBA1* model (Henderson et al., 2020). One possible explanation for this decrease in grip strength could be changes in strength and rigidity, two well-established symptoms of PD (David et al., 2012). Although these symptoms are established deficits in PD, the pathophysiology is still ill-defined and might involve regions outside of the SNpc (Magrinelli et al., 2016; David et al., 2012). Further characterization of this phenotype to explore the pathological underpinnings of this deficit would be interesting.

The second model investigated in this study – the aSyn PFF model – was selected as it displays nigrostriatal degeneration and aSyn pathology on which to overlay the *GBA1* D409V mutation. In this model, injection of murine aSyn PFFs resulted in the expected nigrostriatal degeneration and aSyn pathology (Luk et al., 2012, 2016; Fares et al., 2016; Abdelmotilib et al., 2017; Froula et al., 2019). Similar to the *GBA1* D409V KI×mThy1-hSNCA mouse, the *GBA1* D409V KI mouse did not display worsened nigrostriatal degeneration as compared to WT mice following injection of aSyn PFFs (Fig. 6). Following aSyn PFF injection, *GBA1* D409V KI mice did not display increased pS129 aSyn pathology at 90 DPI as compared to WT mice, but a trend towards more pS129 aSyn-positive cells was observed at 180 DPI (Fig. 5). Although not statistically significant, this is similar to the increase in pS129 aSyn pathology observed in the *GBA1* D409V KI×mThy1-hSNCA mouse at 12 months of age (Fig. 1). Future studies could analyze longer timepoints to determine whether differences are observed at 6 months post-injection.

Although previous models have shown that aSyn overexpression can further decrease GCase activity (Fishbein et al., 2014; Kim et al., 2018), injection of pathogenic aSyn PFFs in this study did not result in a further decrease in GCase activity or increase in GSL levels in the *GBA1* D409V KI mice as compared to aSyn monomer-injected controls (Fig. 7). This could be due to the drastic decrease in GCase activity in homozygous *GBA1* D409V KI mice causing a floor effect, or the fact that the analyses were done within hemibrain samples rather than analyzing structures independently. Future studies combining the aSyn PFF model with heterozygous *GBA1* D409V KI mice and performing GCase and GSL analyses within individual brain structures would be a good step to determine whether there is any effect of aSyn pathology on GCase activity and substrate levels in the *GBA1* D409V KI mouse.

Although the genetic linkage of *GBA1* mutations and PD in humans is clear, the lack of nigrostriatal degeneration in these two models could provide evidence for either unique biology within the mouse that intervenes and masks the role of decreased GCase activity in exacerbating PD-related pathology, and/or a mutation-dependent toxic gain-of-function effect of various *GBA1* mutations in driving pathology. Previous work *in vitro* demonstrated a *GBA1* mutation-dependent increase in aSyn levels within two rodent immortalized cell lines that was independent of GCase protein levels (Cullen et al., 2011). Evidence from that study indicates a greater impact of the L444P mutation as compared to the N370S mutation (Cullen et al., 2011), corresponding to observations in the Taguchi et al. (2017) study that the L444P mutation increased aSyn pathology and shortened lifespan, whereas the N370S mutation did not demonstrate these effects, even though GCase activity and substrate levels were similar between the mutations. Interestingly, however, the Cullen et al. (2011) study suggested that the *GBA1* D409V and D409H mutations resulted in the greatest accumulation of aSyn as compared to other mutations. Although we did observe an increase in pS129 aSyn in the *GBA1* D409V KI×mThy1-hSNCA mouse model, there was no resulting nigrostriatal dysfunction, indicating that there might be mechanisms required for degeneration outside of pathological aSyn phosphorylation that are not strongly driven by the D409V mutation.

When analyzing the various models used to investigate the relationship between aSyn pathology and GCase deficiency, an interesting pattern arises that might explain the lack of exacerbated nigrostriatal degeneration in the two aSyn models used within this study. Fishbein et al. (2014) and Kim et al. (2018) used a synuclein model overexpressing human A53T aSyn – a mutation with a very high aggregation propensity (Li et al., 2002; Lazaro et al., 2014) – and reported the most significant pathology resulting from the combined aSyn and GCase pathologies. Taguchi et al. (2017) used a model overexpressing human A30P aSyn – a mutation with an intermediate aggregation propensity (Lazaro et al., 2014) – and reported a more moderate pathological phenotype. Finally, Migdalska-Richards et al. (2017) and our *GBA1* D409V KI×Thy1 aSyn model used overexpression of WT human aSyn – less aggregation prone as compared to A53T and A30P (Lazaro et al., 2014) – and saw the lowest amount of relative pathology. Furthermore, Fishbein et al. (2014) compared the effects of the *GBA1* L444P heterozygous mutation on a human A53T aSyn overexpression model versus a human WT aSyn overexpression model, and found pS129 aSyn pathology within the context of the A53T mutation, but not wild-type synuclein. This leads to the possibility that overexpression of an aggregation-prone form of aSyn might be required to drive the degenerative effects of decreased GCase activity on the nigrostriatal system within the mouse model system.

Analyzed as a whole, characterization of these two models – the *GBA1* D409V KI×mThy1-hSNCA mouse and the aSyn PFF model in *GBA1* D409V KI mice – provides insight into the relationship between aSyn pathology and GCase deficiency in PD models. The robust increase in pS129 aSyn in the *GBA1* D409V KI×mThy1-hSNCA mouse as compared to the lack of increased aSyn pathology in the aSyn PFF model at 90 DPI and minimal, non-significant increase in pS129 aSyn at 180 DPI is interesting. A mechanism for these differences could be found by comparing the various synuclein models used. The aSyn pathology in the *GBA1* D409V KI×mThy1-hSNCA mouse is driven by overexpression of human wild-type aSyn, whereas the aSyn pathology in the PFF model is driven by changes in endogenous mouse aSyn following recombinant murine aSyn PFF injection (Luk et al., 2012). Therefore, the *GBA1* D409V mutation might require overexpression of aSyn rather than pathological changes in endogenous aSyn. Supporting evidence for this possibility can be found when examining other studies investigating the GCase-aSyn relationship in dual-hit models as well as all other studies that employ aSyn overexpression models (Fishbein et al., 2014; Taguchi et al., 2017; Migdalska-Richards et al., 2017; Kim et al., 2018; Henderson et al., 2020).

Interestingly, a recent study using aSyn PFFs and CBE suggests that models with high levels of aSyn pathology are less susceptible to exacerbated aSyn pathology from the decreased GCase activity. Henderson et al. (2020) noted differences in CBE-exacerbated pathology in primary neuron cultures from different brain regions with varying levels of aSyn expression. Analysis of aSyn pathology *in vivo* in mice following aSyn PFF±CBE treatment revealed a similar, yet subtle, brain region-specific difference. Extensive analysis identified that these effects were due to extant levels of aSyn pathology, whereby regions with less aSyn pathology displayed greater impact of CBE treatment (Henderson et al., 2020). The effect was confirmed in neuronal cultures from heterozygous *GBA1* D409V KI mice. Therefore, the lack of exacerbated aSyn pathology in the *GBA1* D409V KI mice treated with aSyn PFFs might be due to the dose of PFFs used. Future studies using lower doses of aSyn PFFs or examining dose response of PFF treatment would help determine whether this were the case.

Taken together, this study sheds light on important aspects of the GCase-aSyn relationship through the characterization of two novel mouse models. The comparison of these two models, as well as placing them in the context of other published studies, emphasizes the importance of model design/selection when studying the relationship between aSyn pathology and GCase deficiency. Some models, such as those using transgenic mice overexpressing aggregation-prone mutant aSyn might have more severe phenotypes than models overexpressing wild-type aSyn. When using the aSyn PFF model, sub-pathological doses of aSyn PFFs might be required to observe the GCase effect. Finally, selection of the specific mutation in *GBA1* could impact the extent to which synuclein pathology or nigrostriatal degeneration is exacerbated. Therefore, the model used to study the aSyn-GCase interaction should be selected after consideration of the type of pathology desired and the relevance to human cases of PD.

## MATERIALS AND METHODS

### Establishment of experimental cohorts (studies 1 and 2)

#### Study 1: *GBA1* D409V KI×mThy1-hSNCA mice

All breeding, husbandry and maintenance occurred at The Jackson Laboratory in a standard barrier mouse pathogen-free vivarium in individually ventilated cages with aspen bedding. Mice had *ad libitum* access to water and food and were fed a standard LabDiet^®^ 5K52 formulation with 6% fat; water was acidified. The colonies were maintained in a 12 h/12 h light/dark cycle. All animal monitoring and procedures met guidelines and regulations of federal, state and local agencies, in addition to the Association for the Assessment and Accreditation of Laboratory Animal Care International (AAALAC).

A double mutant mouse was established through breeding of the *GBA1* D409V KI mouse previously described in Polinski et al. (2021) with the mThy1-hSNCA mouse described in Choi et al. (2020). Briefly, a D427V point mutation was inserted within the locus of the murine *Gba1* gene (NCBI Gene ID 14466), located on chromosome 3. The D427V mutation corresponds to the D409V mutation in the mature GCase protein, as described by Xu et al. (2003). Thereby, the nomenclature of this line is referred to as the *GBA1* D409V KI mouse (C57Bl/6N-Gba^tm1.1^Mjff/J mouse; Strain No. 019106, The Jackson Laboratory, Bar Harbor, ME, USA). Heterozygous *GBA1* D409V KI mice were bred with hemizygous C57BL/6N-Tg(Thy1-SNCA)15Mjff/J mice (mThy1-hSNCA; Strain No. 017682, The Jackson Laboratory) at The Jackson Laboratory. The mice were pooled at weaning based on date of birth into *n*=20 males per cage. Genotyping was performed prior to 8 weeks of age using standard PCR genotyping procedures, and mice were rehoused based on genotype at *n*=10/genotype/weaning cage. Cage density was reduced to *n*=5/cage as mice aged or showed signs of hair loss. If signs of aggression were detected, males were separated and individually housed as needed. Of note, the double mutant line is now available at The Jackson Laboratory (Strain No. 029124).

From the weaning colonies, experimental cohorts of male homozygous *GBA1* D409V KI×homozygous mThy1-hSNCA mice were reserved and aged to 4, 8 and 12 months of age (*n*=15/timepoint). In addition, separate cohorts of male wild-type C57BL/6NJ mice (WT C57BL/6; Strain No. 005304, The Jackson Laboratory), mThy1-hSNCA mice (Strain No. 017682, The Jackson Laboratory) and *GBA1* D409V KI mice (Strain No. 019106, The Jackson Laboratory) were also reserved and aged to 4, 8 and 12 months of age (*n*=15/timepoint/line). For these experimental cohorts, dates of birth ranged no more than ±3 days for the 4 month cohort, and ±7 days for the 8 and 12 month cohorts.

When the target ages were reached, the mice were shipped live to Charles River Labs (formerly WIL Research) in Ashland, OH, USA. Following a brief acclimation period of 1 week, viability observations for moribundity and mortality were performed twice daily. Body weight was assessed twice weekly and on the day of euthanasia. Cohorts were examined in a functional observational battery once approximately 2 weeks prior to euthanasia, which included locomotor activity. More details on the functional observational battery (FOB) and behavioral testing are provided below.

#### Study 2: *GBA1* D409V KI mice for aSyn PFF injections

PsychoGenics (Paramus, NJ, USA) obtained heterozygous *GBA1* D409V KI (Strain No. 019106, The Jackson Laboratory) and C57Bl/6NJ mice (Strain No. 005304, The Jackson Laboratory) and confirmed genotype via standard PCR using a tail clip. All animals were housed in polycarbonate cages with filter tops in groups of four to five animals. The room temperature was maintained between 20°C and 23°C with a relative humidity around 50%, with a 12 h/12 h light/dark cycle. Chow and water were provided *ad libitum*, with wet chow placed on the cage floor if needed due to animal health condition. Health and survival monitoring were performed twice daily. All animal monitoring and procedures met guidelines and regulations of federal, state and local agencies, in addition to the AAALAC.

To generate a cohort of homozygous *GBA1* D409V KI mice and WT littermate controls, one heterozygous *GBA1* D409V KI mouse was bred with two C57Bl/6 WT females for up to 3 days. Males were then introduced to a new pair of C57Bl/6 females for up to 3 days. A total of five male heterozygous *GBA1* D409V KI mice were used for this initial breeding round. Offspring mice were genotyped at 10 days of age using standard PCR. Once reaching sexual maturity, heterozygous breeding pairs were established to produce the experimental cohort of 92 homozygous *GBA1* D409V KI mice and 92 WT littermates (mixed gender). The experimental cohort was housed until 8 weeks of age when aSyn monomer/PFF injections were performed.

### Injection of aSyn PFFs in the *GBA1* D409V KI mice (study 2)

#### Generation of aSyn PFFs

Mouse aSyn PFFs were kindly provided by Dr Kelvin Luk at the University of Pennsylvania. Purification of recombinant, full-length mouse aSyn protein and *in vitro* fibril assembly was performed as previously described (Luk et al., 2012; Polinski et al., 2018). Briefly, recombinant full-length wild-type mouse aSyn was purified from *Escherichia coli* BL21-RIL cells expressing aSyn constructs from the PRK172 expression vector. Purification methods included gel filtration and ion-exchange chromatography, with purity confirmed by Coomassie Blue staining. PFFs were generated from the monomeric protein by diluting the starting monomer to 360 mM (5 mg/ml) in sterile Dulbecco's PBS (D-PBS; pH 7.0; Mediatech) and incubating with constant agitation (1000 rpm at 37°C) for 7 days in an Eppendorf Thermomixer C with ThermoTop. Successful PFF formation was verified by sedimentation assay and Thioflavin T (EMD Millipore, Burlington, MA, USA) binding.

On the day of surgery, aliquots of the pre-aggregated aSyn were thawed at room temperature and diluted to 2 mg/ml using sterile D-PBS in a sterile microcentrifuge tube. Samples were sonicated when completely thawed using a probe sonicator (Branson Sonifier 150). The sonicator was cleaned using 10% SDS for a duration of 10 pulses (0.5 s per pulse) and then dried. Samples were sonicated by lowering the sonicator into the 0.5 ml Eppendorf tube and providing 20 pulses at 0.5 s each with a 0.5 s inter-pulse interval. After a 30 s rest to allow cooling and frothing to subside, another 20 pulses were performed in the same fashion. This was then followed by another 30 s rest period and another final 20 pulses. The sonicated PFF tube was then sealed with paraffin and carried to the surgical suite to begin inoculations. Immediately prior to use, the tube containing the aSyn PFF solution was flicked gently to mix contents.

#### Surgical method

In total, 184 mice, sex balanced, were randomly assigned and injected unilaterally in the striatum with aSyn PFFs (*n*=46/genotype) or aSyn monomers (*n*=46/genotype). Mice were anesthetized with isoflurane and injected stereotaxically in the right hemisphere with recombinant mouse aSyn PFFs or monomers (2 µg/µl). A single-needle insertion into the right forebrain was used to target the inoculum to the dorsal neostriatum (+0.2 mm relative to bregma, +2.0 mm from midline). A 10 µl Hamilton syringe (Hamilton, Reno, NV, USA) was used for the injection with infusion at a rate of 0.1 µl/min (2.5 µl total). The needle was left in place for ≥5 min following administration. All mice received analgesia both pre- and post-operatively. Buprenex (0.1 mg/kg, subcutaneous) was administered on the day of surgery once the mouse was fully awake. If the mouse showed signs of distress in the 24 h post-surgery, Buprenex was re-administered at the same dose. Animals were monitored regularly following recovery from surgery and thereafter monitored weekly for body weight and bidaily for survival.

### Necropsy and tissue processing (studies 1 and 2)

#### Study 1: *GBA1* D409V KI×mThy1-hSNCA mice

Mice were split into two cohorts for necropsy and analysis: group 1 for neurochemistry (*n*=6/genotype) and group 2 for histology (*n*=9/genotype). Group 1 mice were euthanized with an overdose of sodium pentobarbital [50-100 mg/kg, intraperitoneal (ip)] and intracardially perfused with saline *in situ*. Brains were rapidly harvested and the striatum microdissected, weighed and fresh frozen in individual Eppendorf tubes for later analysis by ultra-high performance liquid chromatography with tandem mass spectrometry (UHPLC/MS/MS) as described below. Group 2 mice were euthanized with an overdose of sodium pentobarbital (50-100 mg/kg, ip) and intracardially perfused with ambient-temperature sodium cacodylate to clear the blood, followed by ice-cold sodium cacodylate-based 4% paraformaldehyde (PFA) for fixation. Following decapitation, brains remained in the skull for 24 h at 4°C in sodium cacodylate-based 4% PFA. Whole brains were harvested, post-fixed for 24 h in sodium cacodylate-based 4% PFA and 24 h in PBS at 4°C, and shipped to NeuroScience Associates (Knoxville, TN, USA) for histological and stereological analysis, as described below.

#### Study 2: *GBA1* D409V KI mice for aSyn PFF injections

Mice were split into two cohorts for necropsy and analysis: group 1 for neurochemistry and GCase/GSL analysis (*n*=12/genotype/injectate) and group 2 for histology (*n*=11/genotype/injectate). Group 1 mice were euthanized with an overdose of sodium pentobarbital (50-100 mg/kg, ip) and intracardially perfused with saline *in situ*. Brains were rapidly harvested and the striatum microdissected, weighed, flash frozen in liquid nitrogen, and stored at −80°C in individual Eppendorf tubes for later analysis by UHPLC/MS/MS as described below. The remaining brain tissue from the striatal microdissections and the caudal brain tissue was hemisected in the sagittal plan to separate hemispheres, and flash frozen in liquid nitrogen prior to storage at −80°C and shipment to Amicus Therapeutics for GCase activity analysis and GSL measurements, as described below. Group 2 mice were euthanized with an overdose of sodium pentobarbital (50-100 mg/kg, ip) and intracardially perfused with ambient temperature sodium cacodylate to clear the blood, followed by ice-cold sodium cacodylate-based 4% PFA for fixation. Following decapitation, brains remained in the skull for 24 h at 4°C in sodium cacodylate-based 4% PFA. Whole brains were harvested, post-fixed for 24 h in sodium cacodylate-based 4% PFA and for 24 h in PBS at 4°C, and shipped to NeuroScience Associates for histological and stereological analysis, as described below. For all animals, the left hemisphere was designated the control hemisphere as it was contralateral to injection, and the right hemisphere was designated the experimental hemisphere as it was ipsilateral to the injection.

### Neurochemistry for striatal neurotransmitters (studies 1 and 2)

#### Study 1: *GBA1* D409V KI×mThy1-hSNCA mice

Neurochemistry for dopamine, its metabolites and other relevant neurotransmitters was performed by Charles River Bioanalytical Chemistry Department (formerly WIL Research) according to the Laboratory Method for the Analysis of DA, 5-HT, DOPAC, HVA, 5-HIAA and norepinephrine (NE) in mouse brain homogenate by liquid chromatography with tandem mass spectrometry (LC/MS/MS) (Lab Method No: 784005A.MT), as described in Polinski et al. (2021). Briefly, frozen microdissected striata from group 1 (neurochemistry) mice were placed on wet ice to partially thaw and homogenized in 0.1% formic acid Milli-Q water (FA-MQ) at a volume of 9:1 (v/w). Samples were then centrifuged at 4000 ***g*** at 4°C prior to immediate extraction.

For sample extraction, a 96-well plate on wet ice was used. A volume of 0.025 ml FA-MQ was added to each well designated as blank, and 0.025 ml of Internal Standard Working Solution was added to each well designated to contain a calibrator, quality control, blank with external standard or experimental sample. A volume of 0.175 ml chilled FA-MQ was added to each sample well. The plate was covered and vortex-mixed for 2 min at 1500 rpm followed by centrifugation at 3500 rpm for 5 min. For dilution-filtration sample extraction, 0.200 ml of the supernatant was loaded into a Whatman UNIFILTER 96-well Microplate (7720-7236, GE Healthcare Life Sciences, Marlborough, MA, USA) using a multichannel pipette, and pushed through the filter to a clean collection plate. The plate was centrifuged at 3500 rpm for 5 min at 4°C and moved to the sample compartment of the LC instrument for analysis.

Detection of neurochemicals was performed using UHPLC/MS/MS (API 4000, ESI+) for DA, NE, 5-HT and 5-HIAA, and UHPLC/MS/MS (API 4000, ESI−) for DOPAC and HVA. A Waters Acquity^®^ UPLC instrument was equipped with autosampler and a Restek PFP Propyl, 50×2.1 mm, 1.9 µm particle size column (9419252, Restek, Bellefonte, PA, USA) for positive ion-mode analysis, or a Waters UPLC HSS T3, 50×2.1 mm, 1.8 µm particle size column (176001131 or 186003538, Waters) for negative ion-mode analysis. Positive-mode sample injection was performed prior to negative-mode analysis. Run time was 4 min at 4°C. For positive ion analysis, an Applied Biosystems/MDS Sciex API 4000™ triple quadrupole instrument with a Turbo Spray, positive-ion interface, and multiple reaction monitoring scan mode was used with the following settings: curtain gas 20.0, gas setting 60.0, ionization voltage 5500 V, temperature 550°C, collision gas setting 8.00, entrance potential 10.0, and interface heater on. For negative ion analysis, an Applied Biosystems/MDS Sciex API 4000™ with a Turbo Spray, negative-ion interface, and multiple reaction monitoring scan mode was used with the following conditions: curtain gas 20.0, gas setting 60.0, ionization voltage −4200 V, temperature 550°C, collision gas setting 8.00, entrance potential −10.0 and interface heater on. A two-way ANOVA test was used for statistical analysis with Tukey post hoc tests for significant effects of genotype or age. GraphPad Prism Software (version 7.05, San Diego, CA, USA) was used for statistical analysis and graphing.

#### Study 2: *GBA1* D409V KI mice for aSyn PFF injections

Neurochemistry for the aSyn PFF model in *GBA1* D409V KI mice was performed by Psychogenics. Frozen microdissected striata from group 1 (neurochemistry) mice were homogenized in 0.2 M perchloric acid including 100 µM EDTA-2Na (1:10, w/v) at 0°C. After 10 min on ice, homogenates were centrifuged for 15 min at 3000 ***g*** at 4°C. The supernatant was collected and mixed with 0.4 M sodium acetate buffer (pH 3.0; 1:2, v/v) and filtered through a 0.22 µm centrifugal filter (4 min, 14,000 ***g***, 4°C). Filtrates were stored at −80°C prior to HPLC analysis.

Monoamines DA and 5-HT, as well as their metabolites DOPAC, HVA and 5-HIAA, were determined with HPLC. Briefly, the HPLC system consists of a HTEC500 apparatus (Eicom, Kyoto, Japan) and a CMA/200 refrigerated microsampler (CMA Microdialysis, Stockholm, Sweden) equipped with a 20 µl loop operating at 4°C. The potential of the glassy carbon working electrode was set at 450 V relative to the Ag/AgCl reference electrode. Separation was achieved using a 200×2.0 mm Eicompak CAX column (Eicom) protected with a guard column CAX-GC2/20 (Eicom). The mobile phase was a mixture of methanol and 0.1 M sodium acetate solution (pH 3.5; 16:84, v/v) containing octanesulfonic acid sodium salt (210 mg/l) and EDTA (5 mg/l). All chromatograms were recorded and integrated using the computerized digital acquisition system clarity (DataApex, Prague, Czech Republic). Repeated measures two-way ANOVA test was used for statistical analysis with Sidak post hoc tests for significant effects of group (genotype×injectate) or hemisphere (within subject variable). GraphPad Prism Software (version 7.05) was used for statistical analysis and graphing.

### Immunohistochemistry and unbiased stereology (studies 1 and 2)

#### Tissue processing

Mice from group 2 in both studies were processed for histology at NeuroScience Associates. Brains were treated overnight with 20% glycerol and 2% dimethylsulfoxide to prevent freeze artifacts prior to being multiply embedded in gelatin matrices using MultiBrain^®^ Technology. After curing, blocks were rapidly frozen by immersion in isopentane chilled to −70°C. Frozen blocks were mounted on the freezing stage of an AO 860 sliding microtome and sectioned in the coronal plane at 40 µm. All sections were collected sequentially in 24 cups per block filled with antigen preserve solution (49% PBS at pH 7.0, 50% ethylene glycol, 1% polyvinyl pyrrolidone). Brain sections were stored at −20°C until staining.

#### Immunohistochemistry

Brain sections were processed and stained free floating as described in previous reports (Dave et al., 2014; Polinski et al., 2021). Briefly, all incubation solutions from blocking serum onwards utilized a vehicle of TBS containing 0.3% Triton X-100 for permeabilization. All rinses were performed with TBS. For chromogen staining, endogenous peroxidase activity was blocked by 0.9% hydrogen peroxide treatment. Following TBS rinses, the sections were immunostained with a primary antibody overnight at room temperature. After primary incubation, sections were rinsed and incubated in a secondary antibody for 2 h at room temperature. The following antibody combinations were used for fluorescent staining: chicken anti-GFAP primary antibody (1:1500; CPCA-GFAP, EnCor, Gainesville, FL, USA) with donkey anti-chicken Alexa Fluor 488 (1:500; 703-545-155, Jackson Immunoresearch, West Grove, PA, USA); goat anti-Iba-1 primary antibody (1:1500; ab5076, Abcam) with donkey anti-goat Alexa Fluor 647 (1:500; A21447, ThermoScientific, Waltham, MA, USA); mouse anti-phospho-serine 129 alpha-synuclein (1:15,000; 010-26841, Wako, Tokyo, Japan) with donkey anti-mouse Straptavidin Cy3 550 (1:500; 016-160-084, Jackson Immunoresearch); rabbit anti-TH primary antibody (1:1500; P40101-0, Pel-Freez, Rogers, AR, USA) with donkey anti-rabbit Alexa Fluor 555 (1:500; A31572, ThermoScientific). The following antibody combinations were used for chromogen staining: rabbit anti-TH primary antibody (1:6000; P40101-0, Pel-Freez) with goat anti-rabbit IgG-biotin (1:238; BA-1000, Vector Laboratories, Burlingame, CA, USA); mouse anti-phospho-serine 129 alpha-synuclein (1:15,000; 010-26841, Wako) with horse anti-mouse IgG-biotin (1:238; BA-2001, Vector Laboratories). Thionin was used for the Nissl stain in study 2.

For chromogen staining, sections were incubated with an avidin-biotin-HRP complex (Vectastain Elite ABC kit, Vector Laboratories) for 1 h at room temperature followed by rinses and treatment with diaminobenzidine tetrahydrochloride (DAB) and 0.0015% hydrogen peroxide. DAB-stained tissue was mounted on gelatinized glass slides, air dried, dehydrated in alcohol, cleared in xylene and coverslipped with Permount. For fluorescent staining, sections were washed twice in 1 min increments in 50% ethanol, followed by several washes in TBS. Sections were mounted on SuperFrost Plus slides and coverslipped with Vectashield mounting medium (Vector Laboratories). Slides with chromogen-stained tissue were scanned using a TissueScope LE120 from Huron Digital Pathology at 20× resolution (0.40 μm/pixel). Slides with fluorescently stained tissue were scanned using an Olympus VS200 Research Slide Scanner with fluorescence imaging capabilities. Slides were scanned with an Olympus 20× X-line extended apochromat objective (0.274 μm/pixel). Exposure settings were kept constant across all scanned slides. All brightfield and fluorescent images were shared via Concentriq, an online image-sharing and management platform developed by Proscia (Philadelphia, PA, USA).

#### Stereological estimates

The unbiased stereology method used in this study has been described in detail in previous publications (Healy-Stoffel et al., 2012, 2014; Polinski et al., 2021). Briefly, a Nikon Eclipse E800 microscope connected with an IMI Tech Color Digital Video Camera was operated using an Advanced Scientific Instrumentation MS-2000 motorized stage input into a Dell Precision 650 Server, and a high-resolution plasma monitor was used in tandem with the Stereologer software package (Stereology Resource Center, Inc.) to estimate neuronal number. For the estimation of neuronal number, every eighth section containing the SNpc (−4.56 to −6.60 mm from bregma) was selected using systemic-random sampling. The section sampling fraction (ssf) was 1/6 (West et al., 1991). Each coronal section was visualized first at 4× magnification to outline the region of interest in reference to the stereotaxic atlas of the mouse brain. Systematic random grids for counting frame were applied by the software. In each counting frame, TH-immunoreactive neurons, Nissl-positive neurons or pS129 aSyn-positive neurons were counted if nucleoli were in focus (not touching the exclusion lines or within the 3 µm guard zones) at approximately 3600× magnification using a 100× objective and 1.4 aperture oil immersion lens. The optical disector probe (Sterio, 1984) was used in combination with optical sectioning in the *z*-axis (West et al., 1991). After every sample coronal brain section was analyzed, the Stereologer software package (Stereology Resource Center, Inc.) estimated the total number of TH/Nissl/pS129 aSyn-positive neurons in the interest area (N) by multiplying the number of counted neurons (ΣQ) with the reciprocals of the section sampling fraction (ssf), the area sampling fraction (asf) and the section thickness sampling fraction (stsf). The average number of slides per animal varied from five to seven, and the coefficient of error (CE) was capped at 0.15 with the actual mean value of 0.96.

For TH stereology in study 1, a two-way ANOVA test was used for statistical analysis with Tukey post hoc tests for significant effects of genotype or age. For TH and Nissl stereology in study 2, a repeated measures two-way ANOVA test was used for statistical analysis with Sidak post hoc tests for significant effects of group (genotype×injectate) or hemisphere (within subject variable). For pS129 aSyn stereology in study 2, unpaired two-tailed *t*-tests were performed for effect of genotype. GraphPad Prism Software (version 7.05) was used for statistical analysis and graphing.

#### Staining intensity quantification

Blinded images of pS129 aSyn DAB-stained sections were uploaded to the HALO image analysis platform from Indica Labs (v3.1.1076.301). One level of substantia nigra (compacta and reticulata) was traced and analyzed for each animal (densitometric area quantification of positive pS129 aSyn signal). Generated data included positive signal area, percent area and average optical density. Data were exported and organized into an Excel spreadsheet. A two-way ANOVA test was used for statistical analysis with Tukey post hoc tests for significant effects of genotype or age. GraphPad Prism Software (version 7.05) was used for statistical analysis and graphing.

Blinded images of GFAP/Iba1/TH/Hoechst 33342-labeled sections were uploaded to the HALO image analysis platform from Indica Labs (v3.1.1076.301). One level of substantia nigra (compacta and reticulata) was traced and analyzed for each animal (densitometric area quantification of positive signal for GFAP, Iba1 and TH). Generated data included positive signal area, percent area, average optical density and colocalization. Data were exported and organized into an Excel spreadsheet. A two-way ANOVA test was used for statistical analysis with Tukey post hoc tests for significant effects of genotype or age. GraphPad Prism Software (version 7.05) was used for statistical analysis and graphing.

Blinded images of GFAP/Iba1/pS129 aSyn/Hoechst 33342-labeled sections were uploaded to the HALO image analysis platform from Indica Labs (v3.1.1076.301). One level of substantia nigra (compacta only) was traced and analyzed for each animal (densitometric area quantification of positive signal for GFAP, Iba1 and pS129 aSyn). Generated data included positive signal area, percent area, average optical density and colocalization. Data were exported and organized into an Excel spreadsheet. Unpaired two-tailed *t*-tests were performed for Iba1 and GFAP staining intensity at 90 and 180 DPI to analyze the effect of genotype.

For all animals and stain sets, unilateral analysis was performed.

### Behavioral analysis of *GBA1* D409V KI×mThy1-hSNCA mice (study 1)

#### Functional observational battery (FOB)

The noninvasive FOB – a collection of assessments used to detect gross functional deficits – was performed weekly on mice to evaluate general behavioral phenotypes. The FOB consists of a series of tests categorized into various domain observations: home cage (posture, convulsions, tremors, eyelid closure, etc.), handling (ease of removal from cage, lacrimation, salivation, piloerection, respiratory rate, mucous secretions, muscle tone, etc.), open field (time to first step, rearing, mobility, grooming, gait/gait score, convulsions, tremors, arousal, bizarre/stereotypic behavior, etc.), sensory (approach, touch, startle response, tail pinch, olfactory orientation, pupil and eyeblink response, forelimb/hindlimb extension, air righting reflex, etc.), neuromuscular (hindlimb extensor strength, forelimb grip strength, hindlimb grip strength, hindlimb foot splay, etc.) and physiological (catalepsy, body temperature and body weight) (Irwin, 1968).

#### Locomotor activity – total activity and total ambulation

Mice were placed in an automated motor activity box constructed from Plexiglas^®^ with the dimensions 48.3×26.7×20.3 cm. Activity was quantified over a 60 min period. Basic and fine movements were recorded and the sum of both was reported as total activity. Ambulation in the *x-* and *y*-planes were recorded and the sum of both was reported as total ambulation. For analysis, total activity and total ambulation were calculated by averaging the measures from the 12 intervals and presenting the activity counts in six 10 min blocks. Assessments excluded the first interval to account for acclimation to the environment. A two-way ANOVA test was used for statistical analysis with Tukey post hoc tests for significant effects of genotype or age. GraphPad Prism Software (version 7.05) was used for statistical analysis and graphing.

#### Forelimb and hindlimb grip strength

Grip strength was measured using a device similar to the one described by Meyer et al. (1979). The mouse was allowed to grip a T-shaped bar with its forepaws and pulled back gently along a platform until its grip was broken. As the backward locomotion continued, the animal's hind paws reached a T-shaped rear limb grip bar, which it grasped and was then forced to release by continued pulling. A Mark-10 series EG digital force gauge (Mark-10 Corporation, Copiague, NY, USA) was used to record the maximum strain required to break forelimb and hindlimb grip. The average of three valid measurements was taken as the animal's score for each grip strength measure. Grip strength is reported in grams (g). A two-way ANOVA test was used for statistical analysis with Tukey post hoc tests for significant effects of genotype or age. GraphPad Prism Software (version 7.05) was used for statistical analysis and graphing.

### Assessment of glucocerebrosidase activity and glycosphingolipid levels (study 2)

#### CBE/4-MUG GCase activity method

Portions of brain and liver tissue from mice in group 2 were processed for GCase activity at Amicus. Samples were homogenized in GCase enzyme buffer (McIlvaine citrate/phosphate pH 5.2 containing 0.25% sodium taurocholate and 0.1% Triton X-100). The protein concentration was determined with the bicinchoninic acid (BCA) assay once per sample from each independent sample of homogenate using bovine serum albumin (BSA) as a standard. GCase activity was determined in triplicate from two independent samples of each homogenate in a 30 min reaction at 37°C in GCase enzyme buffer supplemented to 300 µM N-(n-butyl)deoxygalactonojirimycin and with or without the covalent GCase inhibitor CBE using 4-MUG as the substrate. CBE-inhibitable GCase activity was converted to nmoles of 4-MUG released by comparison with a 4-MUG standard curve run with each assay. These levels were normalized to protein mass and hour. A two-way ANOVA test was used for statistical analysis with Sidak post hoc tests for significant effects of genotype and injectate. GraphPad Prism Software (version 7.05) was used for statistical analysis and graphing.

#### Glycosphingolipid analysis

The remaining unprocessed tissue from samples sent to Amicus were processed for GSL analyses of GlcCer and GlcSph. Samples were homogenized in water, and the protein concentration was determined with the BCA assay in triplicate using BSA as a standard. Lipid extraction included the addition of the appropriate internal standard for GlcCer and for GlcSph, and was performed using solid phase extraction. A liquid chromatography method was employed that separates the more predominant galactosylceramide (GalCer) and galactosylsphingosine (GalSph) epimers from GlcCer and GlcSph observed in brain tissue. Isocratic conditions were used with a hydrophilic interaction liquid chromatography (HILIC) silica column and seven GlcCer isoforms were monitored: C16:0, C18:0, C20:0, C22:0, C23:0, C24:0 and C24:1. The levels of GlcCer and GlcSph were normalized to tissue weight. A two-way ANOVA test was used for statistical analysis with Sidak post hoc tests for significant effects of genotype and injectate. GraphPad Prism Software (version 7.05) was used for statistical analysis and graphing.

### Statistical analyses

All statistical tests are reported in the above sections for each experiment. In all cases, the experimental unit is a single animal and data are represented as mean±s.e.m. Statistical analyses and graphing were performed using the GraphPad Prism Software (version 7.05). Outliers were identified using the GraphPad Prism Software using the ROUT method. A summary of the statistical test results for each figure can be found in Tables S1 and S2.

## Supplementary Material

Supplementary information
