# Supplementary material for: The GBA1 D409V mutation exacerbates synuclein pathology to differing extents in two alpha-synuclein models
Source: Dis Model Mech. 2022 May 25;15(6):dmm049192. doi: 10.1242/dmm.049192 (PMC9150115; doi:10.1242/dmm.049192)
Supplement: Supplementary information [file dmm-15-049192-s1.pdf]

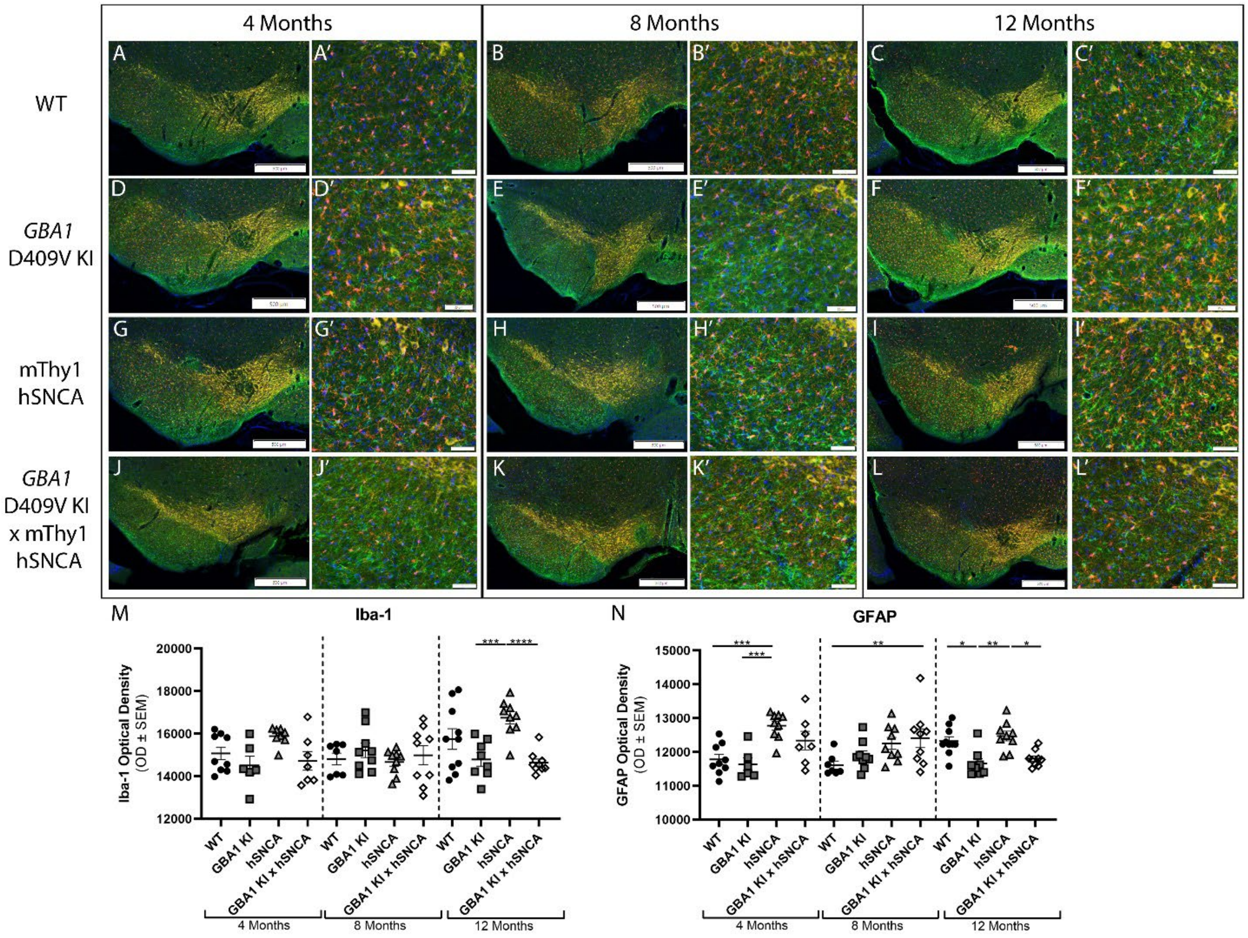

Fig. S1. Microgliosis and astrogliosis in the substantia nigra is not exacerbated by the *GBA1* D409V mutation in the mThy1-hSNCA mouse. Representative images of immunohistochemical staining for astrocytes using GFAP (green), microglia using Iba-1 (red), dopaminergic neurons using tyrosine hydroxylase (TH; yellow), and nuclei using DAPI (blue) in C57Bl/6 wild-type (WT; A-C), *GBA1* D409V KI (D-F), mThy1-hSNCA (G-I), and *GBA1* D409V KI x mThy1-hSNCA (J-L) mice at 4, 8, and 12 months of age (n=9/group). Primed images are higher magnification images taken at 20x. (M-N) Quantitation of staining intensity reveal no increase in microgliosis (M) or astrogliosis (N) in *GBA1* D409V KI x mThy1-hSNCA mice any age. Significant differences from a two-way ANOVA with Tukey *post hoc* tests are reported as follows: *$p < 0.05$, **$p < 0.01$, ***$p < 0.001$, ****$p < 0.0001$. Abbreviations: WT, wild-type; *GBA1*, gene encoding human glucocerebrosidase; KI, knock-in; OD, optical density; Iba-1, Ionized calcium binding adaptor molecule 1; GFAP, Glial fibrillary acidic protein; pS129 aSyn, alpha-synuclein phosphorylated at serine 129; SEM, standard error of the mean.

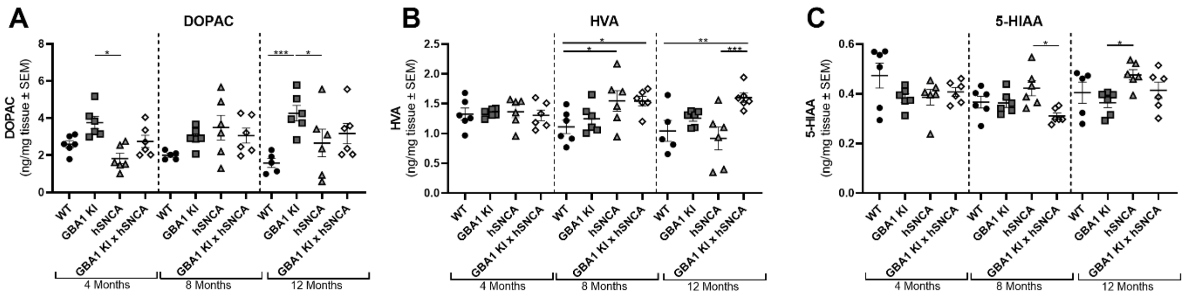

Fig. S2. DOPAC and HVA are increased in the striatum of the *GBA1* D409V KI x mThy1-hSNCA mouse at 12 months of age. Striatal DOPAC (A), HVA (B), and 5-HIAA (C) assessed by UHPLC/MS/MS in striatal tissue in C57Bl/6 wild-type (WT), *GBA1* D409V KI, mThy1-hSNCA, and *GBA1* D409V KI x mThy1-hSNCA mice at 4, 8, and 12 months of age (n=6/group). (A) DOPAC levels are slightly increased in the *GBA1* D409V KI mouse as compared to WT mice at 12 months of age. (B) HVA levels are increased in the *GBA1* D409V KI x mThy1-hSNCA mouse as compared to WT mice at 8 and 12 months of age and mThy1-hSNCA mice at 12 months of age. (C) 5-HIAA levels are unchanged in the *GBA1* D409V KI x mThy1-hSNCA mouse as compared to WT mice at all ages. Significant differences from a two-way ANOVA with Tukey *post hoc* tests are indicated as follows: **p* < 0.05, ***p* < 0.01, ****p* < 0.001. Abbreviations: aSyn, alpha-synuclein; *GBA1*, gene encoding human glucocerebrosidase; WT, wild type; KI, knock-in; mo, month old; ng, nanogram; mg, milligram; DOPAC, 3,4-Dihydroxyphenylacetic acid; HVA, homovanillic acid; 5-HIAA, 5-hydroxyindoleacetic acid; SEM, standard error of the mean.

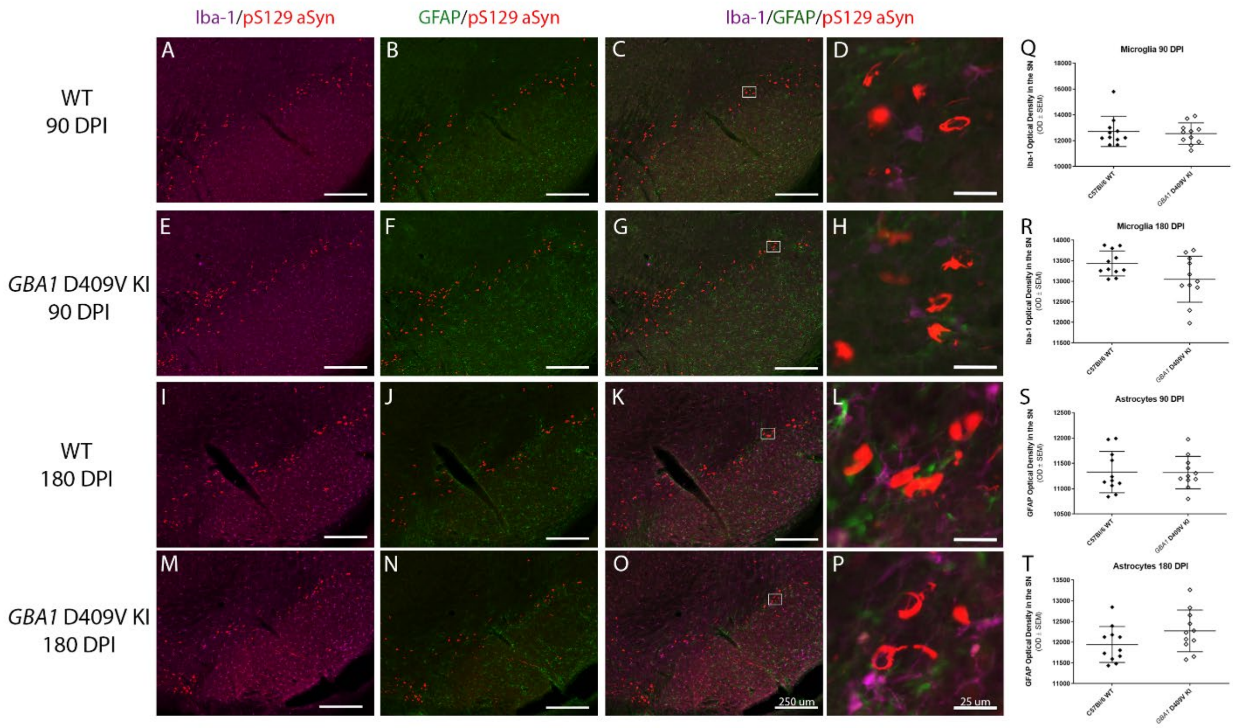

Fig. S3. Microgliosis and astrogliosis in the substantia nigra are not worsened by the *GBA1* D409V mutation following injection of alpha-synuclein preformed fibrils. Representative images of immunohistochemical staining for microglia using Iba-1 (purple), astrocytes using GFAP (green) and alpha-synuclein phosphorylated at S129 (pS129 aSyn; red) in C57Bl/6 wild-type (WT; A-D, I-L) and *GBA1* D409V KI (E-H, M-P) mice at 90 and 180 days post aSyn preformed fibril (PFF) injection. Panels D, H, L, and P are higher magnification images taken using the 40x objective lens of the area outlined in panels C, G, K, and O, respectively. (Q-T) Quantification of staining intensity confirms no increase in microgliosis (Q) or astrogliosis (R) in *GBA1* D409V KI as compared to WT mice at 90 or 180 days post-injection. No significant differences were identified by unpaired t-test. Abbreviations: WT, wild-type; *GBA1*, gene encoding human glucocerebrosidase; KI, knock-in; OD, optical density; Iba-1, Ionized calcium binding adaptor molecule 1; GFAP, Glial fibrillary acidic protein; pS129 aSyn, alpha-synuclein phosphorylated at serine 129; SEM, standard error of the mean; DPI, days post-injection.

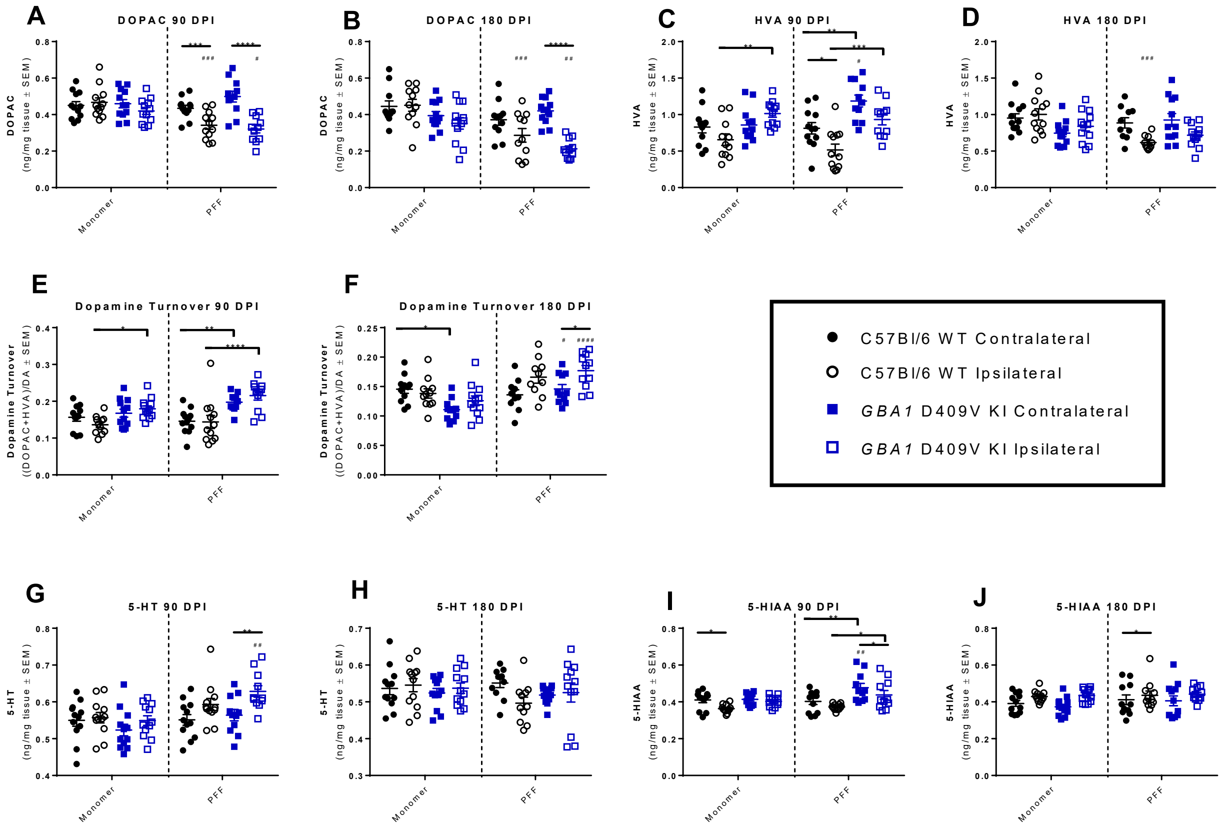

Fig. S4. The *GBA1* D409V mutation does not exacerbated striatal neurochemical deficits induced by alpha-synuclein preformed fibril administration. Striatal DOPAC (A-B), HVA (C-D), 5-HT (G-H), and 5-HIAA (I-J) assessed by UHPLC/MS/MS and dopamine turnover calculations (E-F) in striatal tissue in C57Bl/6 wild-type (WT) and *GBA1* D409V KI mice at 90 and 180 days following intrastriatal injection of alpha-synuclein (aSyn) monomer or preformed fibrils (PFF; n=11-12/group). (A-B) aSyn PFF administration significantly reduced DOPAC levels at 90 (A) and 180 (B) days post injection (DPI), but this decrease was not impacted by genotype. (C-D) The *GBA1* D409V KI mouse displayed some increases in HVA at 90 DPI in both the injected and uninjected hemisphere (C), but these increases did not persist in the 180 DPI cohort (D) and the magnitude change in HVA upon aSyn PFF administration was not impacted by genotype. (E-F) Calculations of dopamine turnover reveal some genotype-related increases in the *GBA1* D409V KI mice in the 90 DPI (E) cohort which do not persist in the 180 DPI cohort (F); aSyn PFF administration has little effect on dopamine turnover. (G-H) Serotonin (5-HT) is slightly increased in the aSyn PFF-injected hemisphere of *GBA1* D409V KI mice at 90 DPI (G), but levels are similar to those observed in WT mice and the increase does not persist to 180 DPI (H). (I-J) The *GBA1* D409V KI mouse displayed some increases in 5-HIAA at 90 DPI in both the ipsilateral and contralateral hemisphere

following aSyn PFF injection (I), but these increases did not persist in the 180 DPI cohort (J). Significant differences between injectate (monomer vs PFF) are indicated as follows: $^{\#}p < 0.05$, $^{\#\#}p < 0.01$, $^{\#\#\#}p < 0.001$, $^{\#\#\#\#}p < 0.0001$. Significant differences between hemisphere or genotype from a repeated measures two-way ANOVA with Sidak *post hoc* tests are denoted as follows: $*p < 0.05$, $**p < 0.01$, $***p < 0.001$, $****p < 0.0001$ (bar indicates hemispheric differences, bracket denotes genotype differences). Abbreviations: WT, wild type; *GBA1*, gene encoding human glucocerebrosidase; KI, knock-in; PFF, preformed fibril; DPI, days post injection; ng, nanogram; mg, milligram; DOPAC, 3,4-Dihydroxyphenylacetic acid; HVA, homovanillic acid; 5-HT, 5-hydroxytryptamine; 5-HIAA, 5-hydroxyindoleacetic acid; SEM, standard error of the mean; DPI, days post-injection.

Table S1. Statistical Analyses for Study 1 using *GBA1* D409V KI x mThy1-hSNCA Mice

Figure	Two-Way ANOVA Statistical Results	Tukey Post Hoc Test Results (Genotype)	Tukey Post Hoc Test Results (Age)
Figure 1A<br><br>Nigral pS129 aSyn (optical density)	Genotype: F (3, 91) = 17.64; P<0.0001<br>Age: F (2, 91) = 1.421; P=0.2469	4<br>WT vs. GBA1: 0.9988<br>WT vs. mThy1: 0.9879<br>WT vs. GBA1xmThy1: <0.0001<br>GBA1 vs. mThy1: 0.9983<br>GBA1 vs. GBA1xmThy1: <0.0001<br>mThy1 vs. GBA1xmThy1: <0.0001<br>8<br>WT vs. GBA1: 0.9723<br>WT vs. mThy1: 0.3937<br>WT vs. GBA1xmThy1: 0.2217<br>GBA1 vs. mThy1: 0.6613<br>GBA1 vs. GBA1xmThy1: 0.4430<br>mThy1 vs. GBA1xmThy1: 0.9851<br>12<br>WT vs. GBA1: 0.9348<br>WT vs. mThy1: 0.2474<br>WT vs. GBA1xmThy1: 0.0001<br>GBA1 vs. mThy1: 0.5877<br>GBA1 vs. GBA1xmThy1: 0.0013<br>mThy1 vs. GBA1xmThy1: 0.0450	WT C57Bl/6<br>4 vs. 8: 0.8070<br>4 vs. 12: 0.7209<br>8 vs. 12: 0.9848<br>GBA1 D409V KI<br>4 vs. 8: 0.9993<br>4 vs. 12: 0.9999<br>8 vs. 12: 0.9998<br>mThy1-hSNCA<br>4 vs. 8: 0.4075<br>4 vs. 12: 0.3149<br>8 vs. 12: 0.9832<br>GBA1 D409V KI x mThy1-hSNCA<br>4 vs. 8: 0.0013<br>4 vs. 12: 0.3834<br>8 vs. 12: 0.0390
Figure 2A<br><br>Body Weight (g)	Genotype: F (3, 163) = 12.21; P<0.0001<br>Age: F (2, 163) = 39.57; P<0.0001	4<br>WT vs. GBA1: 0.9921<br>WT vs. mThy1: 0.9979<br>WT vs. GBA1xmThy1: 0.1006<br>GBA1 vs. mThy1: 0.9675<br>GBA1 vs. GBA1xmThy1: 0.0572<br>mThy1 vs. GBA1xmThy1: 0.1471<br>8<br>WT vs. GBA1: 0.7683<br>WT vs. mThy1: 0.0526<br>WT vs. GBA1xmThy1: 0.0911<br>GBA1 vs. mThy1: 0.0029<br>GBA1 vs. GBA1xmThy1: 0.0061<br>mThy1 vs. GBA1xmThy1: 0.9959<br>12<br>WT vs. GBA1: 0.0876<br>WT vs. mThy1: 0.1447<br>WT vs. GBA1xmThy1: 0.1645<br>GBA1 vs. mThy1: 0.9933<br>GBA1 vs. GBA1xmThy1: <0.0001<br>mThy1 vs. GBA1xmThy1: 0.0002	WT C57Bl/6<br>4 vs. 8: 0.0271<br>4 vs. 12: <0.0001<br>8 vs. 12: 0.0087<br>GBA1 D409V KI<br>4 vs. 8: 0.1446<br>4 vs. 12: 0.0026<br>8 vs. 12: 0.2728<br>mThy1-hSNCA<br>4 vs. 8: <0.0001<br>4 vs. 12: 0.0034<br>8 vs. 12: 0.2085<br>GBA1 D409V KI x mThy1<br>4 vs. 8: 0.0388<br>4 vs. 12: <0.0001<br>8 vs. 12: 0.0148

Disease Models & Mechanisms • Supplementary information

Figure 2B<br><br>Forelimb Grip Strength (g)	Genotype: F (3, 162) = 55.41; P<0.0001<br>Age: F (2, 162) = 70.02; P<0.0001	4<br>WT vs. GBA1: 0.9920<br>WT vs. mThy1: 0.0219<br>WT vs. GBA1xmThy1: 0.9359<br>GBA1 vs. mThy1: 0.0109<br>GBA1 vs. GBA1xmThy1: 0.9902<br>mThy1 vs. GBA1xmThy1: 0.0050<br>8<br>WT vs. GBA1: 0.5905<br>WT vs. mThy1: <0.0001<br>WT vs. GBA1xmThy1: 0.8523<br>GBA1 vs. mThy1: 0.0111<br>GBA1 vs. GBA1xmThy1: 0.1745<br>mThy1 vs. GBA1xmThy1: <0.0001<br>12<br>WT vs. GBA1: <0.0001<br>WT vs. mThy1: <0.0001<br>WT vs. GBA1xmThy1: <0.0001<br>GBA1 vs. mThy1: 0.9632<br>GBA1 vs. GBA1xmThy1: <0.0001<br>mThy1 vs. GBA1xmThy1: <0.0001	WT C57Bl/6<br>4 vs. 8: 0.0103<br>4 vs. 12: 0.0523<br>8 vs. 12: <0.0001<br>GBA1 D409V KI<br>4 vs. 8: <0.0001<br>4 vs. 12: 0.0042<br>8 vs. 12: 0.4996<br>mThy1-hSNCA<br>4 vs. 8: <0.0001<br>4 vs. 12: 0.9423<br>8 vs. 12: <0.0001<br>GBA1 D409V KI x mThy1-hSNCA<br>4 vs. 8: 0.0239<br>4 vs. 12: <0.0001<br>8 vs. 12: <0.0001
Figure 2C<br><br>Hindlimb Grip Strength (g)	Genotype: F (3, 161) = 31.81; P<0.0001<br>Age: F (2, 167) = 1.233; P=0.2941	4<br>WT vs. GBA1: 09867<br>WT vs. mThy1: 0.0621<br>WT vs. GBA1xmThy1: 0.1434<br>GBA1 vs. mThy1: 0.1483<br>GBA1 vs. GBA1xmThy1: 0.0754<br>mThy1 vs. GBA1xmThy1: <0.0001<br>8<br>WT vs. GBA1: 0.6955<br>WT vs. mThy1: 0.9667<br>WT vs. GBA1xmThy1: 0.8656<br>GBA1 vs. mThy1: 0.3912<br>GBA1 vs. GBA1xmThy1: 0.9885<br>mThy1 vs. GBA1xmThy1: 0.5888<br>12<br>WT vs. GBA1: <0.0001<br>WT vs. mThy1: 0.0004<br>WT vs. GBA1xmThy1: <0.0001<br>GBA1 vs. mThy1: 0.0060<br>GBA1 vs. GBA1xmThy1: <0.0001<br>mThy1 vs. GBA1xmThy1: <0.0001	WT C57Bl/6<br>4 vs. 8: 0.5361<br>4 vs. 12: 0.7383<br>8 vs. 12: 0.1798<br>GBA1 D409V KI<br>4 vs. 8: 0.9304<br>4 vs. 12: <0.0001<br>8 vs. 12: <0.0001<br>mThy1-hSNCA<br>4 vs. 8: 0.6081<br>4 vs. 12: 0.6627<br>8 vs. 12: 0.1670<br>GBA1 D409V KI x mThy1-hSNCA<br>4 vs. 8: 0.0431<br>4 vs. 12: 0.0044<br>8 vs. 12: <0.0001

Disease Models & Mechanisms • Supplementary information

Figure 2D<br><br>Open Field Activity (s)	Genotype: F (3, 163) = 9.218; P<0.0001<br>Age: F (2, 163) = 18.70; P<0.0001	4<br>WT vs. GBA1: 0.9983<br>WT vs. mThy1: 0.1980<br>WT vs. GBA1xmThy1: 0.0518<br>GBA1 vs. mThy1: 0.2858<br>GBA1 vs. GBA1xmThy1: 0.0378<br>mThy1 vs. GBA1xmThy1: <0.0001<br>8<br>WT vs. GBA1: 0.0178<br>WT vs. mThy1: 0.4935<br>WT vs. GBA1xmThy1: 0.0802<br>GBA1 vs. mThy1: 0.4056<br>GBA1 vs. GBA1xmThy1: 0.9421<br>mThy1 vs. GBA1xmThy1: 0.7540<br>12<br>WT vs. GBA1: 0.6278<br>WT vs. mThy1: 0.1760<br>WT vs. GBA1xmThy1: 0.6071<br>GBA1 vs. mThy1: 0.8428<br>GBA1 vs. GBA1xmThy1: 0.0714<br>mThy1 vs. GBA1xmThy1: 0.0058	WT C57Bl/6<br>4 vs. 8: 0.0050<br>4 vs. 12: 0.0419<br>8 vs. 12: 0.7699<br>GBA1 D409V KI<br>4 vs. 8: 0.9992<br>4 vs. 12: 0.0022<br>8 vs. 12: 0.0021<br>mThy1-hSNCA<br>4 vs. 8: 0.9746<br>4 vs. 12: 0.0292<br>8 vs. 12: 0.0161<br>GBA1 D409V KI x mThy1-hSNCA<br>4 vs. 8: 0.0031<br>4 vs. 12: 0.0007<br>8 vs. 12: 0.8952
Figure 2E<br><br>Open Field Ambulation (cm)	Genotype: F (3, 161) = 12.21; P<0.0001<br>Age: F (2, 167) = 32.02; P<0.0001	4<br>WT vs. GBA1: 0.9198<br>WT vs. mThy1: 0.2670<br>WT vs. GBA1xmThy1: 0.0076<br>GBA1 vs. mThy1: 0.0760<br>GBA1 vs. GBA1xmThy1: 0.0517<br>mThy1 vs. GBA1xmThy1: <0.0001<br>8<br>WT vs. GBA1: 0.0038<br>WT vs. mThy1: 0.7247<br>WT vs. GBA1xmThy1: 0.0959<br>GBA1 vs. mThy1: 0.0780<br>GBA1 vs. GBA1xmThy1: 0.6708<br>mThy1 vs. GBA1xmThy1: 0.5735<br>12<br>WT vs. GBA1: 0.9879<br>WT vs. mThy1: 0.0959<br>WT vs. GBA1xmThy1: 0.6904<br>GBA1 vs. mThy1: 0.1893<br>GBA1 vs. GBA1xmThy1: 0.4816<br>mThy1 vs. GBA1xmThy1: 0.0040	WT C57Bl/6<br>4 vs. 8: 0.0042<br>4 vs. 12: 0.0063<br>8 vs. 12: 0.9971<br>GBA1 D409V KI<br>4 vs. 8: 0.9088<br>4 vs. 12: 0.0003<br>8 vs. 12: 0.0010<br>mThy1-hSNCA<br>4 vs. 8: 0.9272<br>4 vs. 12: 0.0010<br>8 vs. 12: 0.0034<br>GBA1 D409V KI x mThy1-hSNCA<br>4 vs. 8: 0.0002<br>4 vs. 12: <0.0001<br>8 vs. 12: 0.4936

Figure 3A<br><br>Striatal Dopamine (ng/mg tissue)	Genotype: F (3, 59) = 1.369; P=0.2611<br>Age: F (2, 59) = 13.66; P<0.0001	4<br>WT vs. GBA1: 0.9381<br>WT vs. mThy1: 0.0172<br>WT vs. GBA1xmThy1: 0.7179<br>GBA1 vs. mThy1: 0.0738<br>GBA1 vs. GBA1xmThy1: 0.9640<br>mThy1 vs. GBA1xmThy1: 0.1987<br>8<br>WT vs. GBA1: >0.9999<br>WT vs. mThy1: 0.0102<br>WT vs. GBA1xmThy1: 0.0614<br>GBA1 vs. mThy1: 0.0110<br>GBA1 vs. GBA1xmThy1: 0.0653<br>mThy1 vs. GBA1xmThy1: 0.9016<br>12<br>WT vs. GBA1: 0.9802<br>WT vs. mThy1: 0.0067<br>WT vs. GBA1xmThy1: 0.9548<br>GBA1 vs. mThy1: 0.0011<br>GBA1 vs. GBA1xmThy1: 0.7796<br>mThy1 vs. GBA1xmThy1: 0.0194	WT C57Bl/6<br>4 vs. 8: 0.8955<br>4 vs. 12: 0.9684<br>8 vs. 12: 0.9812<br>GBA1 D409V KI<br>4 vs. 8: 0.9940<br>4 vs. 12: 0.9965<br>8 vs. 12: 0.9815<br>mThy1-hSNCA<br>4 vs. 8: 0.8011<br>4 vs. 12: <0.0001<br>8 vs. 12: <0.0001<br>GBA1 D409V KI x mThy1-hSNCA<br>4 vs. 8: 0.1336<br>4 vs. 12: 0.3771<br>8 vs. 12: 0.0047
Figure 3B<br><br>Striatal Dopamine Turnover (DOPAC+HVA/DA)	Genotype: F (3, 58) = 2.548; P=0.06463<br>Age: F (2, 58) = 12.67; P<0.0001	4<br>WT vs. GBA1: 0.8257<br>WT vs. mThy1: 0.1721<br>WT vs. GBA1xmThy1: 0.9616<br>GBA1 vs. mThy1: 0.0238<br>GBA1 vs. GBA1xmThy1: 0.5375<br>mThy1 vs. GBA1xmThy1: 0.3914<br>8<br>WT vs. GBA1: 0.8506<br>WT vs. mThy1: 0.9879<br>WT vs. GBA1xmThy1: 0.9838<br>GBA1 vs. mThy1: 0.6702<br>GBA1 vs. GBA1xmThy1: 0.6490<br>mThy1 vs. GBA1xmThy1: >0.9999<br>12<br>WT vs. GBA1: 0.0689<br>WT vs. mThy1: <0.0001<br>WT vs. GBA1xmThy1: 0.0098<br>GBA1 vs. mThy1: 0.0021<br>GBA1 vs. GBA1xmThy1: 0.8606<br>mThy1 vs. GBA1xmThy1: 0.0185	WT C57Bl/6<br>4 vs. 8: 0.8381<br>4 vs. 12: 0.3844<br>8 vs. 12: 0.7019<br>GBA1 D409V KI<br>4 vs. 8: 0.8106<br>4 vs. 12: 0.9244<br>8 vs. 12: 0.5820<br>mThy1-hSNCA<br>4 vs. 8: 0.4665<br>4 vs. 12: <0.0001<br>8 vs. 12: <0.0001<br>GBA1 D409V KI x mThy1-hSNCA<br>4 vs. 8: 0.8989<br>4 vs. 12: 0.0389<br>8 vs. 12: 0.0125

Disease Models & Mechanisms • Supplementary information

Figure 3C<br><br>Striatal 5-HT (ng/mg tissue)	Genotype: F (3, 59) = 3.405; P=0.0233<br>Age: F (2, 59) = 7.889; P=0.0009	4<br>WT vs. GBA1: >0.9999<br>WT vs. mThy1: 0.3127<br>WT vs. GBA1xmThy1: 0.1932<br>GBA1 vs. mThy1: 0.3025<br>GBA1 vs. GBA1xmThy1: 0.1858<br>mThy1 vs. GBA1xmThy1: 0.9924<br>8<br>WT vs. GBA1: 0.8725<br>WT vs. mThy1: 0.8006<br>WT vs. GBA1xmThy1: 0.9996<br>GBA1 vs. mThy1: 0.9988<br>GBA1 vs. GBA1xmThy1: 0.8251<br>mThy1 vs. GBA1xmThy1: 0.7444<br>12<br>WT vs. GBA1: 0.0378<br>WT vs. mThy1: <0.0001<br>WT vs. GBA1xmThy1: 0.0364<br>GBA1 vs. mThy1: 0.0402<br>GBA1 vs. GBA1xmThy1: >0.9999<br>mThy1 vs. GBA1xmThy1: 0.0419	WT C57Bl/6<br>4 vs. 8: 0.4339<br>4 vs. 12: 0.1455<br>8 vs. 12: 0.7502<br>GBA1 D409V KI<br>4 vs. 8: 0.8700<br>4 vs. 12: 0.6612<br>8 vs. 12: 0.3614<br>mThy1-hSNCA<br>4 vs. 8: 0.3441<br>4 vs. 12: <0.0001<br>8 vs. 12: 0.0006<br>GBA1 D409V KI x mThy1-hSNCA<br>4 vs. 8: 0.7791<br>4 vs. 12: 0.0134<br>8 vs. 12: 0.0713
Figure 4<br><br>Nigral Dopamine Neuron Stereology (cell count)	Genotype: F (3, 92) = 1.522; P=0.2140<br>Age: F (2, 92) = 3.846; P=0.0249	4<br>WT vs. GBA1: 0.9958<br>WT vs. mThy1: 0.9979<br>WT vs. GBA1xmThy1: 0.9522<br>GBA1 vs. mThy1: >0.9999<br>GBA1 vs. GBA1xmThy1: 0.9906<br>mThy1 vs. GBA1xmThy1: 0.9840<br>8<br>WT vs. GBA1: 0.9832<br>WT vs. mThy1: 0.7587<br>WT vs. GBA1xmThy1: 0.2029<br>GBA1 vs. mThy1: 0.9258<br>GBA1 vs. GBA1xmThy1: 0.3754<br>mThy1 vs. GBA1xmThy1: 0.7521<br>12<br>WT vs. GBA1: 0.7568<br>WT vs. mThy1: 0.8580<br>WT vs. GBA1xmThy1: 0.5481<br>GBA1 vs. mThy1: 0.9962<br>GBA1 vs. GBA1xmThy1: 0.1114<br>mThy1 vs. GBA1xmThy1: 0.1547	WT C57Bl/6<br>4 vs. 8: 0.8685<br>4 vs. 12: 0.4014<br>8 vs. 12: 0.1748<br>GBA1 D409V KI<br>4 vs. 8: 0.9954<br>4 vs. 12: 0.8794<br>8 vs. 12: 0.9135<br>mThy1-hSNCA<br>4 vs. 8: 0.7882<br>4 vs. 12: 0.7737<br>8 vs. 12: 0.9996<br>GBA1 D409V KI x mThy1-hSNCA<br>4 vs. 8: 0.1426<br>4 vs. 12: 0.0102<br>8 vs. 12: 0.4870

Supplemental Figure 1M<br><br>Nigral Iba1 (optical density)	Genotype: F (3, 88) = 5.367; P=0.0019<br>Age: F (2, 88) = 3.096; P=0.0502	4<br>WT vs. GBA1: 0.6894<br>WT vs. mThy1: 0.3262<br>WT vs. GBA1xmThy1: 0.8897<br>GBA1 vs. mThy1: 0.0510<br>GBA1 vs. GBA1xmThy1: 0.9792<br>mThy1 vs. GBA1xmThy1: 0.1054<br>8<br>WT vs. GBA1: 0.8390<br>WT vs. mThy1: 0.9915<br>WT vs. GBA1xmThy1: 0.9833<br>GBA1 vs. mThy1: 0.6288<br>GBA1 vs. GBA1xmThy1: 0.9589<br>mThy1 vs. GBA1xmThy1: 0.8970<br>12<br>WT vs. GBA1: 0.1701<br>WT vs. mThy1: 0.1270<br>WT vs. GBA1xmThy1: 0.0716<br>GBA1 vs. mThy1: 0.0005<br>GBA1 vs. GBA1xmThy1: 0.9893<br>mThy1 vs. GBA1xmThy1: <0.0001	WT C57Bl/6<br>4 vs. 8: 0.8529<br>4 vs. 12: 0.2968<br>8 vs. 12: 0.1318<br>GBA1 D409V KI<br>4 vs. 8: 0.3540<br>4 vs. 12: 0.8573<br>8 vs. 12: 0.6340<br>mThy1-hSNCA<br>4 vs. 8: 0.0319<br>4 vs. 12: 0.1741<br>8 vs. 12: <0.0001<br>GBA1 D409V KI x mThy1-hSNCA<br>4 vs. 8: 0.8493<br>4 vs. 12: 0.9843<br>8 vs. 12: 0.7268
Supplemental Figure 1N<br><br>Nigral GFAP (optical density)	Genotype: F (3, 89) = 11.84; P<0.0001<br>Age: F (2, 89) = 0.2895; P=0.7494	4<br>WT vs. GBA1: 0.9484<br>WT vs. mThy1: 0.0002<br>WT vs. GBA1xmThy1: 0.1134<br>GBA1 vs. mThy1: 0.0002<br>GBA1 vs. GBA1xmThy1: 0.0567<br>mThy1 vs. GBA1xmThy1: 0.2943<br>8<br>WT vs. GBA1: 0.6674<br>WT vs. mThy1: 0.0522<br>WT vs. GBA1xmThy1: 0.0094<br>GBA1 vs. mThy1: 0.4043<br>GBA1 vs. GBA1xmThy1: 0.1230<br>mThy1 vs. GBA1xmThy1: 0.9096<br>12<br>WT vs. GBA1: 0.0279<br>WT vs. mThy1: 0.9075<br>WT vs. GBA1xmThy1: 0.1026<br>GBA1 vs. mThy1: 0.0053<br>GBA1 vs. GBA1xmThy1: 0.9355<br>mThy1 vs. GBA1xmThy1: 0.0232	WT C57Bl/6<br>4 vs. 8: 0.7802<br>4 vs. 12: 0.0460<br>8 vs. 12: 0.0115<br>GBA1 D409V KI<br>4 vs. 8: 0.5869<br>4 vs. 12: 0.9956<br>8 vs. 12: 0.5965<br>mThy1-hSNCA<br>4 vs. 8: 0.0676<br>4 vs. 12: 0.3958<br>8 vs. 12: 0.6104<br>GBA1 D409V KI x mThy1-hSNCA<br>4 vs. 8: 0.9566<br>4 vs. 12: 0.0815<br>8 vs. 12: 0.0272

Supplemental Figure 2A<br><br>Striatal DOPAC (ng/mg tissue)	Genotype: FF (3, 58) = 7.081; P=0.0004<br>Age: F (2, 58) = 0.2253; P=0.7989	4<br>WT vs. GBA1: 0.2181<br>WT vs. mThy1: 0.5748<br>WT vs. GBA1xmThy1: 0.9938<br>GBA1 vs. mThy1: 0.0104<br>GBA1 vs. GBA1xmThy1: 0.3367<br>mThy1 vs. GBA1xmThy1: 0.4170<br>8<br>WT vs. GBA1: 0.4248<br>WT vs. mThy1: 0.0913<br>WT vs. GBA1xmThy1: 0.3341<br>GBA1 vs. mThy1: 0.8080<br>GBA1 vs. GBA1xmThy1: 0.9981<br>mThy1 vs. GBA1xmThy1: 0.8884<br>12<br>WT vs. GBA1: 0.0004<br>WT vs. mThy1: 0.3287<br>WT vs. GBA1xmThy1: 0.0665<br>GBA1 vs. mThy1: 0.0454<br>GBA1 vs. GBA1xmThy1: 0.2708<br>mThy1 vs. GBA1xmThy1: 0.8269	WT C57Bl/6<br>4 vs. 8: 0.6141<br>4 vs. 12: 0.2550<br>8 vs. 12: 0.8075<br>GBA1 D409V KI<br>4 vs. 8: 0.3813<br>4 vs. 12: 0.6741<br>8 vs. 12: 0.0822<br>mThy1-hSNCA<br>4 vs. 8: 0.0189<br>4 vs. 12: 0.3457<br>8 vs. 12: 0.3509<br>GBA1 D409V KI x mThy1-hSNCA<br>4 vs. 8: 0.8570<br>4 vs. 12: 0.7563<br>8 vs. 12: 0.9815
Supplemental Figure 2B<br><br>Striatal HVA (ng/mg tissue)	Genotype: F (3, 59) = 4.083; P=0.0106<br>Age: F (2, 59) = 2.125; P=0.1285	4<br>WT vs. GBA1: 0.9995<br>WT vs. mThy1: 0.9957<br>WT vs. GBA1xmThy1: 0.9991<br>GBA1 vs. mThy1: 0.9994<br>GBA1 vs. GBA1xmThy1: 0.9945<br>mThy1 vs. GBA1xmThy1: 0.9832<br>8<br>WT vs. GBA1: 0.8294<br>WT vs. mThy1: 0.0426<br>WT vs. GBA1xmThy1: 0.0438<br>GBA1 vs. mThy1: 0.2575<br>GBA1 vs. GBA1xmThy1: 0.2626<br>mThy1 vs. GBA1xmThy1: >0.9999<br>12<br>WT vs. GBA1: 0.5793<br>WT vs. mThy1: 0.8781<br>WT vs. GBA1xmThy1: 0.0085<br>GBA1 vs. mThy1: 0.1574<br>GBA1 vs. GBA1xmThy1: 0.1567<br>mThy1 vs. GBA1xmThy1: 0.0004	WT C57Bl/6<br>4 vs. 8: 0.3793<br>4 vs. 12: 0.2209<br>8 vs. 12: 0.9141<br>GBA1 D409V KI<br>4 vs. 8: 0.8197<br>4 vs. 12: 0.8545<br>8 vs. 12: 0.9975<br>mThy1-hSNCA<br>4 vs. 8: 0.4950<br>4 vs. 12: 0.0199<br>8 vs. 12: 0.0007<br>GBA1 D409V KI x mThy1-hSNCA<br>4 vs. 8: 0.3014<br>4 vs. 12: 0.1654<br>8 vs. 12: 0.9357

Supplemental Figure 2C<br><br>Striatal 5-HIAA (ng/mg tissue)	Genotype: F (3, 59) = 3.065; P=0.0349<br>Age: F (2, 59) = 3.914; P=0.0253	4<br>WT vs. GBA1: 0.1100<br>WT vs. mThy1: 0.1296<br>WT vs. GBA1xmThy1: 0.3393<br>GBA1 vs. mThy1: 0.9998<br>GBA1 vs. GBA1xmThy1: 0.9281<br>mThy1 vs. GBA1xmThy1: 0.9507<br>8<br>WT vs. GBA1: 0.9999<br>WT vs. mThy1: 0.4889<br>WT vs. GBA1xmThy1: 0.5006<br>GBA1 vs. mThy1: 0.4469<br>GBA1 vs. GBA1xmThy1: 0.5438<br>mThy1 vs. GBA1xmThy1: 0.0313<br>12<br>WT vs. GBA1: 0.7679<br>WT vs. mThy1: 0.3068<br>WT vs. GBA1xmThy1: 0.9951<br>GBA1 vs. mThy1: 0.0296<br>GBA1 vs. GBA1xmThy1: 0.5875<br>mThy1 vs. GBA1xmThy1: 0.3944	WT C57Bl/6<br>4 vs. 8: 0.0225<br>4 vs. 12: 0.2201<br>8 vs. 12: 0.6313<br>GBA1 D409V KI<br>4 vs. 8: 0.8720<br>4 vs. 12: 0.8800<br>8 vs. 12: 0.9998<br>mThy1-hSNCA<br>4 vs. 8: 0.6313<br>4 vs. 12: 0.0647<br>8 vs. 12: 0.3607<br>GBA1 D409V KI x mThy1-hSNCA<br>4 vs. 8: 0.0452<br>4 vs. 12: 0.9836<br>8 vs. 12: 0.0296

Disease Models & Mechanisms • Supplementary information

Table S2. Statistical Analyses for Study 2 using aSyn PFF Injection in *GBA1* D409V KI Mice

Figure	Statistical Test	Statistical Analyses Results	Sidak Post Hoc Test Results (Genotype/Injectate)	Sidak Post Hoc Test Results (Hemisphere)
Figure 5I<br><br>90DPI Nigral pS129 aSyn+ Neuron Stereology (cell counts)	Unpaired T-Test	p=0.5383	N/A	N/A
Figure 5J<br><br>180DPI Nigral pS129 aSyn+ Neuron Stereology (cell counts)	Unpaired T-Test	p=0.0756	N/A	N/A
Figure 6A<br><br>90DPI Striatal DA (ng/mg tissue)	Repeated Measures Two Way ANOVA	Group:<br>F (3, 43) = 5.542, P = 0.0026<br>Hemisphere:<br>F (1, 43) = 44.38, P < 0.0001<br>Interaction:<br>F (3, 43) = 14.35, P < 0.0001<br>Within Subject:<br>F (43, 43) = 1.243, P = 0.2397	Contralat WT - Mon v PFF: 0.9742<br>Contralat GBA - Mon v PFF: 0.7781<br>Ipsilat WT - Mon v PFF: < 0.0001<br>Ipsilat GBA - Mon v PFF: < 0.0001<br><br>Contralat Mon - WT v GBA: 0.9482<br>Contralat PFF - WT v GBA: 0.9996<br>Ipsilat Mon - WT v GBA: > 0.9999<br>Ipsilat PFF - WT v GBA: 0.9959	WT Monomer<br>Contra v Ipsi: 0.9906<br>WT PFF<br>Contra v Ipsi: < 0.0001<br>GBA Monomer<br>Contra v Ipsi: 0.9976<br>GBA PFF<br>Contra v Ipsi: < 0.0001
Figure 6B<br><br>180DPI Striatal DA (ng/mg tissue)	Repeated Measures Two Way ANOVA	Group:<br>F (3, 43) = 23.70, P < 0.0001<br>Hemisphere:<br>F (1, 43) = 73.69, P < 0.0001<br>Interaction:<br>F (3, 43) = 27.98, P < 0.0001<br>Within Subject:<br>F (43, 43) = 1.875, P = 0.0211	Contralat WT - Mon v PFF: > 0.9999<br>Contralat GBA - Mon v PFF: 0.1786<br>Ipsilat WT - Mon v PFF: < 0.0001<br>Ipsilat GBA - Mon v PFF: < 0.0001<br><br>Contralat Mon - WT v GBA: 0.5322<br>Contralat PFF - WT v GBA: 0.9991<br>Ipsilat Mon - WT v GBA: 0.3428<br>Ipsilat PFF - WT v GBA: 0.9991	WT Monomer<br>Contra v Ipsi: 0.1549<br>WT PFF<br>Contra v Ipsi: < 0.0001<br>GBA Monomer<br>Contra v Ipsi: 0.1906<br>GBA PFF<br>Contra v Ipsi: < 0.0001
Figure 6C<br><br>180 DPI Nigral TH+ Neuron Stereology (cell counts)	Repeated Measures Two Way ANOVA	Group:<br>F (3, 38) = 8.301, P = 0.0002<br>Hemisphere:<br>F (1, 38) = 71.35, P < 0.0001<br>Interaction:<br>F (3, 38) = 7.446, P = 0.0005<br>Within Subject:<br>F (38, 38) = 1.864, P = 0.0293	Contralat WT - Mon v PFF: 0.3150<br>Contralat GBA - Mon v PFF: 0.9997<br>Ipsilat WT - Mon v PFF: < 0.0001<br>Ipsilat GBA - Mon v PFF: 0.0048<br><br>Contralat Mon - WT v GBA: 0.9999<br>Contralat PFF - WT v GBA: 0.3360<br>Ipsilat Mon - WT v GBA: 0.4821<br>Ipsilat PFF - WT v GBA: > 0.9999	WT Monomer<br>Contra v Ipsi: 0.6318<br>WT PFF<br>Contra v Ipsi: < 0.0001<br>GBA Monomer<br>Contra v Ipsi: 0.0267<br>GBA PFF<br>Contra v Ipsi: < 0.0001
Figure 6D<br><br>180 DPI Nigral Nissl+ Neuron Stereology (cell counts)	Repeated Measures Two Way ANOVA	Group:<br>F (3, 40) = 0.6279, P = 0.6013<br>Hemisphere:<br>F (1, 40) = 13.37, P = 0.0007<br>Interaction:<br>F (3, 40) = 1.164, P = 0.3353<br>Within Subject:	Contralat WT - Mon v PFF: 0.9957<br>Contralat GBA - Mon v PFF: 0.8679<br>Ipsilat WT - Mon v PFF: 0.2405<br>Ipsilat GBA - Mon v PFF: 0.8831<br><br>Contralat Mon - WT v GBA: 0.9378<br>Contralat PFF - WT v GBA: 0.9751<br>Ipsilat Mon - WT v GBA: 0.8735<br>Ipsilat PFF - WT v GBA: 0.9783	WT Monomer<br>Contra v Ipsi: 0.9934<br>WT PFF<br>Contra v Ipsi: 0.0198<br>GBA Monomer<br>Contra v Ipsi: 0.1898<br>GBA PFF<br>Contra v Ipsi: 0.2068

Disease Models & Mechanisms • Supplementary information

		F (40, 40) = 2.020, P = 0.0144		
Figure 7A<br><br>90 DPI GCase Activity (nmol 4MU/hour/mg)	Two Way ANOVA	Genotype:<br>F (1, 43) = 584.5, P < 0.0001<br>Injectate:<br>F (1, 43) = 1.632, P = 0.2083<br>Interaction:<br>F (1, 43) = 0.5630, P = 0.4571	WT<br>Monomer v PFF: 0.9148<br>GBA<br>Monomer v PFF: 0.3000	Monomer<br>WT vs GBA: < 0.0001<br>PFF<br>WT vs GBA: < 0.0001
Figure 7B<br><br>90 DPI GlcCer (ug/g tissue)	Two Way ANOVA	Genotype:<br>F (1, 43) = 0.6504, P = 0.4244<br>Injectate:<br>F (1, 43) = 0.01195, P = 0.9134<br>Interaction:<br>F (1, 43) = 0.2388, P = 0.6276	WT<br>Monomer v PFF: 0.8918<br>GBA<br>Monomer v PFF: 0.9568	Monomer<br>WT vs GBA: 0.5898<br>PFF<br>WT vs GBA: 0.9694
Figure 7C<br><br>90 DPI GlcSph (ug/g tissue)	Two Way ANOVA	Genotype:<br>F (1, 43) = 195.0, P < 0.0001<br>Injectate:<br>F (1, 43) = 0.6471, P = 0.4256<br>Interaction:<br>F (1, 43) = 0.6528, P = 0.4236	WT<br>Monomer v PFF: >0.9999<br>GBA<br>Monomer v PFF: 0.4608	Monomer<br>WT vs GBA: < 0.0001<br>PFF<br>WT vs GBA: < 0.0001
Figure 7D<br><br>180 DPI GCase Activity (nmol 4MU/hour/mg)	Two Way ANOVA	Genotype:<br>F (1, 43) = 358.4, P < 0.0001<br>Injectate:<br>F (1, 43) = 0.00312, P = 0.9557<br>Interaction:<br>F (1, 43) = 0.2601, P = 0.6127	WT<br>Monomer v PFF: 0.9386<br>GBA<br>Monomer v PFF: > 0.9025	Monomer<br>WT vs GBA: < 0.0001<br>PFF<br>WT vs GBA: < 0.0001
Figure 7E<br><br>180 DPI GlcCer (ug/g tissue)	Two Way ANOVA	Genotype:<br>F (1, 43) = 0.8858, P = 0.3519<br>Injectate:<br>F (1, 43) = 0.2916, P = 0.5920<br>Interaction:<br>F (1, 43) = 0.2286, P = 0.6350	WT<br>Monomer v PFF: 0.9988<br>GBA<br>Monomer v PFF: 0.7196	Monomer<br>WT vs GBA: 0.5319<br>PFF<br>WT vs GBA: 0.9363
Figure 7F<br><br>180 DPI GlcSph (ug/g tissue)	Two Way ANOVA	Genotype:<br>F (1, 43) = 340.9, P < 0.0001<br>Injectate:<br>F (1, 43) = 0.07175, P = 0.7901<br>Interaction:<br>F (1, 43) = 0.08880, P = 0.7671	WT<br>Monomer v PFF: 0.9997<br>GBA<br>Monomer v PFF: 0.9025	Monomer<br>WT vs GBA: < 0.0001<br>PFF<br>WT vs GBA: < 0.0001
Supplemental Figure 3Q<br><br>90 DPI Iba1 (optical density)	Unpaired T-Test	p=0.7014	N/A	N/A

Disease Models & Mechanisms • Supplementary information

Supplemental Figure 3R<br><br>180 DPI Iba1 (optical density)	Unpaired T-Test	p=0.0600	N/A	N/A
Supplemental Figure 3S<br><br>90 DPI GFAP (optical density)	Unpaired T-Test	p=0.9516	N/A	N/A
Supplemental Figure 3T<br><br>180 DPI GFAP (optical density)	Unpaired T-Test	p=0.1153	N/A	N/A
Supplemental Figure 4A<br><br>90 DPI Striatal DOPAC (ng/mg tissue)	Repeated Measures Two Way ANOVA	Group:<br>F (3, 43) = 2.767, P = 0.0532<br>Hemisphere:<br>F (1, 43) = 40.97, P < 0.0001<br>Interaction:<br>F (3, 43) = 12.81, P < 0.0001<br>Within Subject:<br>F (43, 43) = 2.746, P = 0.0006	Contralat WT - Mon v PFF: 0.9970<br>Contralat GBA - Mon v PFF: 0.7777<br>Ipsilat WT - Mon v PFF: 0.0007<br>Ipsilat GBA - Mon v PFF: 0.0143<br><br>Contralat Mon - WT v GBA: 0.9998<br>Contralat PFF - WT v GBA: 0.2478<br>Ipsilat Mon - WT v GBA: 0.5710<br>Ipsilat PFF - WT v GBA: 0.9870	WT Monomer<br>Contra v Ipsi: 0.9091<br>WT PFF<br>Contra v Ipsi: 0.0007<br>GBA Monomer<br>Contra v Ipsi: 0.3063<br>GBA PFF<br>Contra v Ipsi: < 0.0001
Supplemental Figure 4B<br><br>180 DPI Striatal DOPAC (ng/mg tissue)	Repeated Measures Two Way ANOVA	Group:<br>F (3, 41) = 6.948, P = 0.0007<br>Hemisphere:<br>F (1, 41) = 21.70, P < 0.0001<br>Interaction:<br>F (3, 41) = 6.567, P = 0.0010<br>Within Subject:<br>F (41, 41) = 1.635, P = 0.0599	Contralat WT - Mon v PFF: 0.3812<br>Contralat GBA - Mon v PFF: 0.9928<br>Ipsilat WT - Mon v PFF: 0.0007<br>Ipsilat GBA - Mon v PFF: 0.0044<br><br>Contralat Mon - WT v GBA: 0.7608<br>Contralat PFF - WT v GBA: 0.8367<br>Ipsilat Mon - WT v GBA: 0.0873<br>Ipsilat PFF - WT v GBA: 0.3633	WT Monomer<br>Contra v Ipsi: 0.9996<br>WT PFF<br>Contra v Ipsi: 0.0802<br>GBA Monomer<br>Contra v Ipsi: 0.6269<br>GBA PFF<br>Contra v Ipsi: < 0.0001
Supplemental Figure 4C<br><br>90 DPI Striatal HVA (ng/mg tissue)	Repeated Measures Two Way ANOVA	Group:<br>F (3, 42) = 14.50, P < 0.0001<br>Hemisphere:<br>F (1, 42) = 6.068, P = 0.0179<br>Interaction:<br>F (3, 42) = 3.303, P = 0.0293<br>Within Subject:<br>F (42, 42) = 0.6730, P = 0.8982	Contralat WT - Mon v PFF: > 0.9999<br>Contralat GBA - Mon v PFF: 0.0145<br>Ipsilat WT - Mon v PFF: 0.7133<br>Ipsilat GBA - Mon v PFF: 0.9707<br><br>Contralat Mon - WT v GBA: > 0.9999<br>Contralat PFF - WT v GBA: 0.0038<br>Ipsilat Mon - WT v GBA: 0.0061<br>Ipsilat PFF - WT v GBA: 0.0009	WT Monomer<br>Contra v Ipsi: 0.4683<br>WT PFF<br>Contra v Ipsi: 0.0458<br>GBA Monomer<br>Contra v Ipsi: 0.5268<br>GBA PFF<br>Contra v Ipsi: 0.1409
Supplemental Figure 4D<br><br>180 DPI Striatal HVA (ng/mg tissue)	Repeated Measures Two Way ANOVA	Group:<br>F (3, 42) = 5.495, P = 0.0028<br>Hemisphere:<br>F (1, 42) = 3.075, P = 0.0868<br>Interaction:<br>F (3, 42) = 3.579, P = 0.0216<br>Within Subject:<br>F (42, 42) = 0.7813, P = 0.7864	Contralat WT - Mon v PFF: 0.9797<br>Contralat GBA - Mon v PFF: 0.9797<br>Ipsilat WT - Mon v PFF: 0.0004<br>Ipsilat GBA - Mon v PFF: 0.6769<br><br>Contralat Mon - WT v GBA: 0.1222<br>Contralat PFF - WT v GBA: 0.9986<br>Ipsilat Mon - WT v GBA: 0.3347<br>Ipsilat PFF - WT v GBA: 0.8612	WT Monomer<br>Contra v Ipsi: 0.9717<br>WT PFF<br>Contra v Ipsi: 0.0460<br>GBA Monomer<br>Contra v Ipsi: 0.7935<br>GBA PFF<br>Contra v Ipsi: 0.1156

Supplemental Figure 4E<br><br>90 DPI Striatal Dopamine Turnover ((DOPAC+HVA)/DA)	Repeated Measures Two Way ANOVA	Group:<br>F (3, 42) = 17.21, P < 0.0001<br>Hemisphere:<br>F (1, 42) = 0.04673, P = 0.8299<br>Interaction:<br>F (3, 42) = 0.9354, P = 0.4321<br>Within Subject:<br>F (42, 42) = 0.6358, P = 0.9269	Contralat WT - Mon v PFF: 0.9855<br>Contralat GBA - Mon v PFF: 0.3116<br>Ipsilat WT - Mon v PFF: 0.9971<br>Ipsilat GBA - Mon v PFF: 0.1358<br><br>Contralat Mon - WT v GBA: 0.9791<br>Contralat PFF - WT v GBA: 0.0088<br>Ipsilat Mon - WT v GBA: 0.0407<br>Ipsilat PFF - WT v GBA: < 0.0001	WT Monomer<br>Contra v Ipsi: 0.6963<br>WT PFF<br>Contra v Ipsi: > 0.9999<br>GBA Monomer<br>Contra v Ipsi: 0.9313<br>GBA PFF<br>Contra v Ipsi: 0.7786
Supplemental Figure 4F<br><br>180 DPI Striatal Dopamine Turnover ((DOPAC+HVA)/DA)	Repeated Measures Two Way ANOVA	Group:<br>F (3, 40) = 12.27, P < 0.0001<br>Hemisphere:<br>F (1, 40) = 8.670, P = 0.0054<br>Interaction:<br>F (3, 40) = 2.311, P = 0.0907<br>Within Subject:<br>F (40, 40) = 0.8497, P = 0.6955	Contralat WT - Mon v PFF: 0.9572<br>Contralat GBA - Mon v PFF: 0.0117<br>Ipsilat WT - Mon v PFF: 0.1038<br>Ipsilat GBA - Mon v PFF: < 0.0001<br><br>Contralat Mon - WT v GBA: 0.0129<br>Contralat PFF - WT v GBA: 0.9490<br>Ipsilat Mon - WT v GBA: 0.8365<br>Ipsilat PFF - WT v GBA: 0.9212	WT Monomer<br>Contra v Ipsi: 0.9538<br>WT PFF<br>Contra v Ipsi: 0.0724<br>GBA Monomer<br>Contra v Ipsi: 0.5572<br>GBA PFF<br>Contra v Ipsi: 0.0442
Supplemental Figure 4G<br><br>90 DPI Striatal 5-HT (ng/mg tissue)	Repeated Measures Two Way ANOVA	Group:<br>F (3, 43) = 4.518, P = 0.0077<br>Hemisphere:<br>F (1, 43) = 14.02, P = 0.0005<br>Interaction:<br>F (3, 43) = 1.679, P = 0.1856<br>Within Subject:<br>F (43, 43) = 1.656, P = 0.0510	Contralat WT - Mon v PFF: > 0.9999<br>Contralat GBA - Mon v PFF: 0.3317<br>Ipsilat WT - Mon v PFF: 0.4513<br>Ipsilat GBA - Mon v PFF: 0.0025<br><br>Contralat Mon - WT v GBA: 0.7708<br>Contralat PFF - WT v GBA: 0.9919<br>Ipsilat Mon - WT v GBA: 0.9994<br>Ipsilat PFF - WT v GBA: 0.4766	WT Monomer<br>Contra v Ipsi: 0.9913<br>WT PFF<br>Contra v Ipsi: 0.1081<br>GBA Monomer<br>Contra v Ipsi: 0.1081<br>GBA PFF<br>Contra v Ipsi: 0.0065
Supplemental Figure 4H<br><br>180 DPI Striatal 5-HT (ng/mg tissue)	Repeated Measures Two Way ANOVA	Group:<br>F (3, 43) = 0.5177, P = 0.6723<br>Hemisphere:<br>F (1, 43) = 0.3630, P = 0.5500<br>Interaction:<br>F (3, 43) = 2.117, P = 0.1121<br>Within Subject:<br>F (43, 43) = 1.250, P = 0.2337	Contralat WT - Mon v PFF: 0.9897<br>Contralat GBA - Mon v PFF: > 0.9999<br>Ipsilat WT - Mon v PFF: 0.2053<br>Ipsilat GBA - Mon v PFF: 0.9958<br><br>Contralat Mon - WT v GBA: 0.9952<br>Contralat PFF - WT v GBA: 0.6605<br>Ipsilat Mon - WT v GBA: 0.9995<br>Ipsilat PFF - WT v GBA: 0.7715	WT Monomer<br>Contra v Ipsi: 0.9894<br>WT PFF<br>Contra v Ipsi: 0.0728<br>GBA Monomer<br>Contra v Ipsi: 0.9584<br>GBA PFF<br>Contra v Ipsi: 0.9964
Supplemental Figure 4I<br><br>90 DPI Striatal 5-HIAA (ng/mg tissue)	Repeated Measures Two Way ANOVA	Group:<br>F (3, 43) = 6.108, P = 0.0015<br>Hemisphere:<br>F (1, 43) = 26.55, P < 0.0001<br>Interaction:<br>F (3, 43) = 1.518, P = 0.2233<br>Within Subject:<br>F (43, 43) = 4.506, P < 0.0001	Contralat WT - Mon v PFF: 0.9995<br>Contralat GBA - Mon v PFF: 0.0198<br>Ipsilat WT - Mon v PFF: 0.9984<br>Ipsilat GBA - Mon v PFF: 0.4407<br><br>Contralat Mon - WT v GBA: > 0.9999<br>Contralat PFF - WT v GBA: 0.0036<br>Ipsilat Mon - WT v GBA: 0.2988<br>Ipsilat PFF - WT v GBA: 0.0134	WT Monomer<br>Contra v Ipsi: 0.0017<br>WT PFF<br>Contra v Ipsi: 0.0676<br>GBA Monomer<br>Contra v Ipsi: 0.8214<br>GBA PFF<br>Contra v Ipsi: 0.0157

Disease Models & Mechanisms • Supplementary information

Supplemental Figure 4J<br><br>180 DPI Striatal 5-HIAA (ng/mg tissue)	Repeated Measures Two Way ANOVA	Group:<br>F (3, 44) = 0.4801, P = 0.6978<br>Hemisphere:<br>F (1, 44) = 15.22, P = 0.0003<br>Interaction:<br>F (3, 44) = 0.6015, P = 0.6175<br>Within Subject:<br>F (44, 44) = 1.996, P = 0.0120	Contralat WT - Mon v PFF: 0.9760<br>Contralat GBA - Mon v PFF: > 0.9999<br>Ipsilat WT - Mon v PFF: > 0.9999<br>Ipsilat GBA - Mon v PFF: > 0.9999<br><br>Contralat Mon - WT v GBA: 0.9641<br>Contralat PFF - WT v GBA: 0.7124<br>Ipsilat Mon - WT v GBA: > 0.9999<br>Ipsilat PFF - WT v GBA: 0.9999	WT Monomer<br>Contra v Ipsi: 0.2488<br>WT PFF<br>Contra v Ipsi: 0.0121<br>GBA Monomer<br>Contra v Ipsi: 0.7047<br>GBA PFF<br>Contra v Ipsi: 0.3035

Disease Models & Mechanisms • Supplementary information
